# Association of Serum 25‐Hydroxyvitamin D With Frailty Across Three Prospective Cohorts: Effect Modification by Physical Activity

**DOI:** 10.1002/fsn3.72589

**Published:** 2026-11-13

**Authors:** Mingkai Li, Yafei Zhao, Chenyang Li, Qinfei Guo, Chenhui Lv, Boqian Zhang, Lushan Xue, Cheng Zhang, Haifeng Zhao

**Affiliations:** ^1^ Nutritional and Food Sciences Research Institute, Department of Nutrition and Food Hygiene, School of Public Health Shanxi Medical University Taiyuan China; ^2^ The Second Clinical Medical College of Shanxi Medical University Taiyuan China; ^3^ MOE Key Laboratory of Coal Environmental Pathogenicity and Prevention, School of Public Health Shanxi Medical University Taiyuan China

**Keywords:** frailty index, incident frailty, physical activity, prospective cohort, serum 25‐hydroxyvitamin D

## Abstract

Vitamin D is a modifiable nutritional factor, but longitudinal evidence on its association with frailty across populations remains limited. We examined the associations of baseline serum 25‐hydroxyvitamin D [25(OH)D] concentrations with frailty index (FI) and incident frailty in three nationally representative cohorts from China, England, and the United States. Data were obtained from the Chinese Longitudinal Healthy Longevity Survey, the English Longitudinal Study of Aging, and the Health and Retirement Study, including 13,164 participants in total. Frailty was assessed using a deficit accumulation‐based FI, and incident frailty was defined as FI ≥ 0.25 among participants who were non‐frail at baseline. Multivariable linear regression, Cox proportional hazards models, and restricted cubic splines were used to evaluate cross‐sectional, longitudinal, and dose–response associations, respectively. Compared with normal 25(OH)D concentrations (> 50 nmol/L), low 25(OH)D concentrations (≤ 50 nmol/L) were consistently associated with higher FI across all three cohorts. Each 10 nmol/L increment in 25(OH)D was significantly associated with lower FI, and non‐linear dose–response relationships were observed in all cohorts. In longitudinal analyses, low 25(OH)D concentrations were associated with higher incident frailty risk in CLHLS (HR 1.76, 95% CI 1.19–2.60) and ELSA (HR 1.27, 95% CI 1.08–1.50), with a similar but non‐significant trend in HRS (HR 1.05, 95% CI 0.86–1.27). Physical activity significantly modified these associations. These findings suggest that lower 25(OH)D concentrations are associated with worse frailty outcomes, and that vitamin D may be a modifiable target for frailty prevention.

## Graphical abstract

We examined associations between circulating 25‐hydroxyvitamin D [25(OH)D] and frailty among 13,164 participants from three population‐based cohorts in China (CLHLS), the United Kingdom (ELSA), and the United States (HRS), including 8651 participants with follow‐up data. Lower 25(OH)D levels were associated with higher frailty index scores and an increased risk of incident frailty. Dose–response analyses suggested nonlinear patterns, and physical activity modified these associations. The findings highlight the relevance of vitamin D status and physical activity to frailty risk in aging populations.

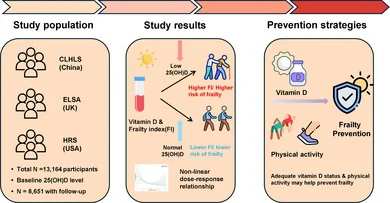

## 1 Introduction

Frailty is an age‐related clinical state characterized by reduced physiological reserve and the accumulation of health deficits, leading to increased vulnerability to stressors (Howlett et al. 2021). It is closely associated with a range of adverse outcomes, including disability, hospitalization, and mortality (Dent et al. 2019). As global population aging accelerates, frailty has become a major challenge to healthy aging, particularly among middle‐aged, and older adults. Among the available approaches to frailty assessment, the frailty index (FI), based on the cumulative health deficit model, provides a sensitive, and continuous measure of frailty severity across the aging spectrum (Mitnitski et al. 2001). Because frailty is dynamic and potentially preventable, growing attention has been directed toward identifying modifiable biological and behavioral factors that may influence its development (Hoogendijk et al. 2019).

Vitamin D, a potentially modifiable nutritional factor, has attracted growing attention for its role in healthy aging (Demay et al. 2024). Beyond its well‐established function in calcium homeostasis and skeletal health, vitamin D has been implicated in several biological pathways relevant to frailty, including skeletal muscle function, inflammation, immune regulation, and metabolic homeostasis (Das et al. 2024; Laird et al. 2014). Observational evidence, including our recent study, has linked lower serum vitamin D concentrations to poorer physical performance, sarcopenia, and a higher likelihood of frailty (Park et al. 2014; Sohl et al. 2013; Zhang et al. 2026; Zheng et al. 2022). Sarcopenia represents an important component in the progression toward frailty, as age‐related losses of muscle mass and strength can impair mobility and reduce physical resilience, thereby accelerating the decline in physiological reserve and increasing vulnerability to stressors (Lynch et al. 2022). These interrelated processes provide a plausible basis for a broader association between vitamin D status and frailty. However, longitudinal evidence on this relationship remains limited, particularly regarding whether the association is consistent across populations with different demographic and health characteristics.

Frailty has emerged as a major global public health challenge in aging societies. Countries with substantial regional and global influence, such as China, England, and the United States, are simultaneously experiencing rapid population aging and an increasing health burden associated with frailty (Fan et al. 2020; Gale et al. 2014; Kurnat‐Thoma et al. 2022). These countries differ markedly in demographic, sociocultural, lifestyle, and healthcare contexts, providing a valuable context in which to assess whether associations between vitamin D status and frailty are consistent across populations. Although previous systematic reviews and meta‐analyses have synthesized evidence from diverse populations, the existing literature remains predominantly cross‐sectional, with relatively limited longitudinal evidence (Khakbaz et al. 2025). In addition, the frameworks used to define and assess frailty have varied across previous studies. Further evaluation across cohorts from different populations using a broadly consistent analytical framework may therefore help clarify the robustness and generalisability of the association between vitamin D status and frailty. Assessing both baseline frailty severity and subsequent incident frailty may also provide a more comprehensive understanding of this association, including whether it varies according to individual or behavioral characteristics.

To address these gaps, we examined the cross‐sectional and longitudinal associations of baseline 25(OH)D concentrations with the FI and incident frailty across three independent population‐based cohorts in China, England, and the United States. We further evaluated potential effect modification and conducted a series of sensitivity analyses to evaluate the robustness of the findings.

## 2 Methods

### 2.1 Study Design and Population

The Chinese Longitudinal Healthy Longevity Survey (CLHLS), the English Longitudinal Study of Aging (ELSA), and the Health and Retirement Study (HRS) are prospective, nationally representative cohort studies conducted in China, England, and the United States, respectively. The three cohorts differed in their target populations due to their respective study designs. Specifically, the CLHLS primarily focused on older adults aged ≥ 65 years, whereas the ELSA and HRS targeted broader populations of middle‐aged and older adults aged ≥ 50 years. The starting ages of the target populations in each cohort were illustrated in Figure 1A. Detailed descriptions of these three cohorts were provided in the Supplementary Methods. For the present study, baseline data were drawn from Wave 6 of the CLHLS (2012), Wave 6 of the ELSA (2012–2013), and Wave 13 of the HRS (2016). The first follow‐up corresponded to Wave 7 of the CLHLS (2014), Wave 7 of the ELSA (2014–2015), and Wave 14 of the HRS (2018). Final outcome ascertainment was based on subsequent follow‐up data from Wave 8 of the CLHLS (2018), Waves 8–9 of the ELSA (2016–2019), and Waves 15–16 of the HRS (2020–2022). The CLHLS was approved by the Biomedical Ethics Committee of Peking University, China (IRB00001052–13074). The ELSA received ethical approval from the London Multi‐Centre Research Ethics Committee (MREC/01/2/91), and the HRS was approved by the University of Michigan Institutional Review Board and the National Institute on Aging (HUM00061128). Written informed consent was obtained from all participants in the three cohorts, and all studies were conducted in accordance with the relevant ethical guidelines and regulations.

**FIGURE 1:**
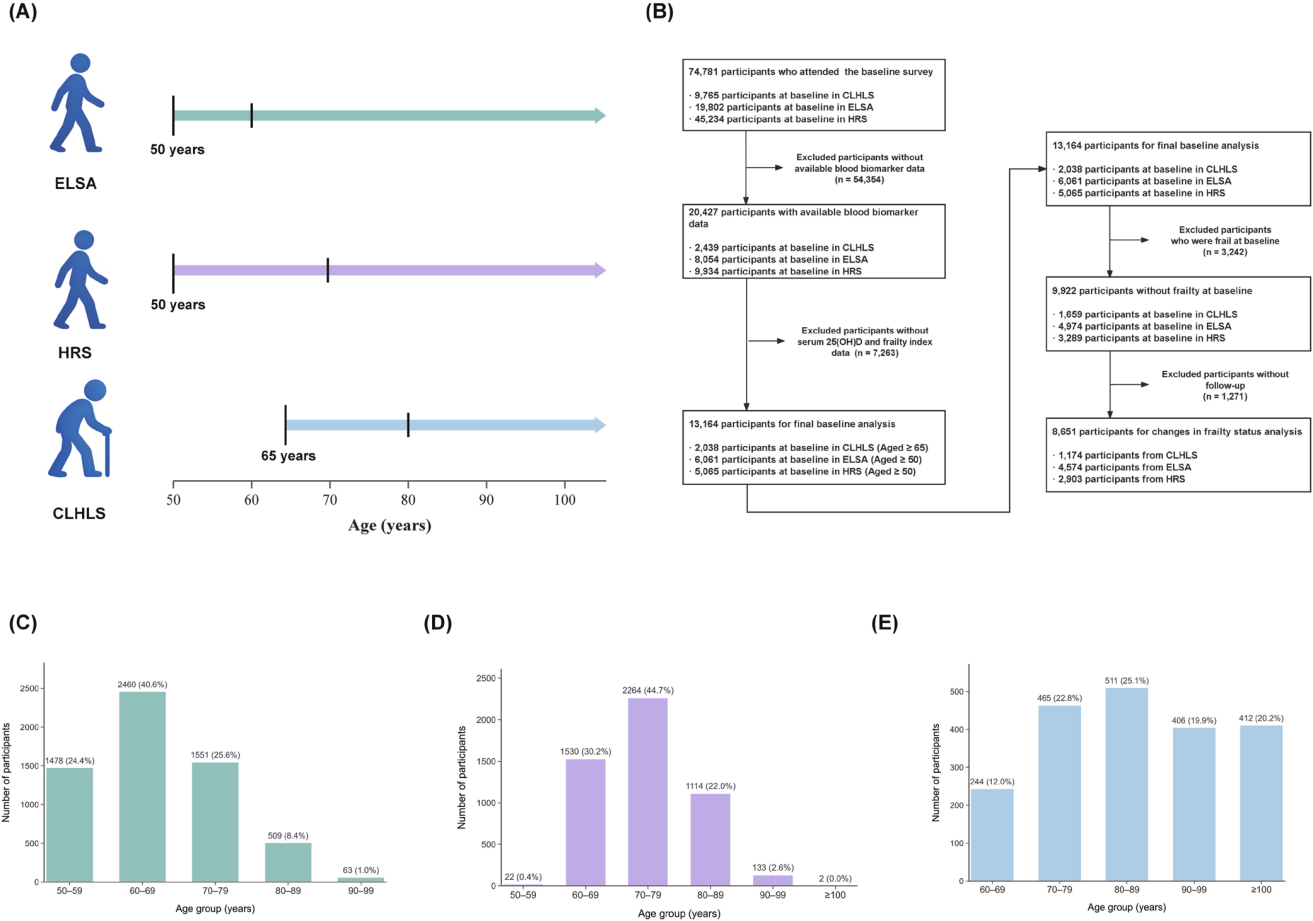
Starting ages of the three cohorts, participant selection, and age distributions of the included participants. (A) Starting ages of the target populations in the CLHLS, ELSA, and HRS. (B) Flowchart of participant selection. (C) Age distributions of participants included in the study from the ELSA. (D) Age distributions of participants included in the study from the HRS. (E) Age distributions of participants included in the study from the CLHLS. 25(OH)D, 25‐hydroxyvitamin D; CLHLS, Chinese Longitudinal Healthy Longevity Survey; ELSA, English Longitudinal Study of Aging; HRS, Health and Retirement Study.

The participant selection process was shown in Figure 1B. Of the 74,781 participants initially identified from the CLHLS, ELSA, and HRS, 54,354 were excluded because they did not undergo blood biomarker testing. We then excluded participants with missing data on serum 25‐hydroxyvitamin D [25(OH)D] or on variables required to calculate the frailty index (FI). In addition, participants younger than 65 years in the CLHLS and younger than 50 years in the ELSA and HRS were excluded. Ultimately, 13,164 participants met the inclusion criteria and were included in the subsequent analyses, comprising 2038 from the CLHLS, 6061 from the ELSA, and 5065 from the HRS. The age distributions of the participants included in the final analytical samples were presented in Figure 1C–E.

### 2.2 Assessment of Vitamin D Status

Vitamin D status was assessed using serum 25(OH)D concentrations, which are widely regarded as the best biomarker of overall vitamin D status (Zerwekh 2008). Serum 25(OH)D was measured by enzyme‐linked immunosorbent assay in the CLHLS, by DiaSorin Liaison immunoassay in the ELSA, and by liquid chromatography–tandem mass spectrometry (LC–MS/MS) in the HRS. Detailed information on the assay methods used in each cohort is provided in the Supplementary Methods.

In the present study, the vitamin D measure in the CLHLS and HRS primarily reflected 25(OH)D3, whereas the measure provided in the ELSA represented total 25(OH)D, defined as the sum of 25(OH)D2 and 25(OH)D3. Because 25(OH)D3 is the predominant endogenous circulating form, and evidence suggests that vitamin D3 is more effective than vitamin D2 in raising and maintaining circulating 25(OH)D concentrations, the use of 25(OH)D3 as the analytical measure is biologically justifiable when 25(OH)D2 and 25(OH)D3 are quantified separately (Cappola et al. 2023; Giustina et al. 2024). Previous studies have shown that 25(OH)D2 is detected infrequently in the general population and has a shorter half‐life than 25(OH)D3 (Jones et al. 2014; Schleicher et al. 2016). Comparative studies and meta‐analyses have further suggested that vitamin D3 is more effective than vitamin D2 in increasing and maintaining circulating 25(OH)D concentrations (Tripkovic et al. 2012). In addition, standardized LC–MS/MS data from National Health and Nutrition Examination Survey (NHANES) showed that 25(OH)D2 was detectable in only 19% of the overall population (Schleicher et al. 2016). Therefore, despite differences in analyte composition and assay platform across cohorts, these measures were used as indicators of vitamin D status. Nevertheless, absolute concentrations should be compared across cohorts with caution. In this study, serum 25(OH)D concentrations ≤ 50 nmol/L were classified as low vitamin D status, whereas concentrations > 50 nmol/L were classified as normal vitamin D status, consistent with previous studies (Holick et al. 2011; Luiz et al. 2022; Zeng et al. 2022).

### 2.3 Assessment of Frailty

Frailty was assessed using the frailty index (FI), constructed according to the standard cumulative deficit approach and analyzed as a continuous variable (Searle et al. 2008). The FI was calculated by dividing the sum of deficit scores by the number of valid indicators included in the calculation, yielding a value ranging from 0 to 1, with higher values indicating greater frailty. Consistent with the cumulative‐deficit framework and previous multi‐cohort studies, the number and composition of individual deficits may vary according to the health measures available within each cohort, while the included deficits generally span multiple health domains, such as chronic diseases, symptoms, disability, physical function, depressive symptoms, and cognition (Stolz et al. 2021; Xiao‐Yu et al. 2025). Previous multi‐cohort studies have shown that a common deficit‐accumulation framework can be applied across aging cohorts even when the individual deficits are not entirely identical (Xiao‐Yu et al. 2025).

Consistent with previous studies, the CLHLS constructed the FI using 40 health‐related indicators (Supplementary Materials Table S1) and only participants with data on at least 30 indicators were included (Huang, Jigeer, et al. 2025). In ELSA and HRS, the FI was constructed using 28 health‐related indicators (He et al. 2024), and participants were required to have complete data for all 28 indicators to be included in the FI analysis (Supplementary Materials Table S2). Although the specific indicators differed across the three cohorts according to the measures available, all cohort‐specific FI was constructed using the same underlying cumulative‐deficit principle. To facilitate comparison of the FI components across cohorts, the specific indicators and their operational definitions in CLHLS, ELSA, and HRS were summarized in Supplementary Materials Table S6. In the present study, FI was further classified as frail (FI ≥ 0.25) or non‐frail (FI < 0.25), and incident frailty was defined as the primary endpoint in longitudinal analyses.

### 2.4 Assessment of Covariates

Covariates included age, sex, education, marital status, place of birth or residence, income, smoking status, drinking status, body mass index (BMI), physical activity, hypertension, and diabetes. To maximize comparability across the CLHLS, ELSA, and HRS cohorts, several variables were harmonized. Marital status was categorized as “Married or partnered” and “Not married.” Education was classified as consistently as possible across cohorts: in the CLHLS, it was grouped according to years of education (0, 1–5, and > 5 years), whereas in the ELSA and HRS it was categorized as less than upper secondary, upper secondary or vocational training, and tertiary education. Smoking status was dichotomized as ever smoking (current or former) versus never smoking, and drinking status as ever drinking (current or former) versus never drinking. Diabetes and hypertension were based on self‐report and coded as yes or no. Weight and height were objectively measured by trained staff, and BMI was calculated as weight in kilograms divided by height in metres squared (kg/m^2^). Previous studies have shown that physical activity is associated with frailty and may also influence 25(OH)D concentrations (Zhang et al. 2024). Taking into account the physical activity information available in each cohort and the need for cross‐cohort comparability, physical activity was classified as either “active” or “inactive.” In the CLHLS, “active” was defined as current or previous participation in exercise, including purposeful fitness activities such as walking, ball games, running, and qigong, whereas “inactive” was defined as never participating in exercise (Huang, Jigeer, et al. 2025). In the ELSA and HRS, “active” was defined as engaging in moderate‐ or vigorous‐intensity physical activity, such as running, swimming, cycling, or aerobics, at least once per week; otherwise, participants were classified as “inactive.” This classification was consistent with previous research (Ammous et al. 2025; Huang, Lai, and Woo 2025).

Given the well‐established seasonal variation in circulating 25(OH)D concentrations (Shoben et al. 2011), season of blood collection was additionally included as a categorical covariate in the multivariable‐adjusted models for the CLHLS and ELSA. This adjustment was applied to both the cross‐sectional analyses of baseline frailty index and the longitudinal analyses of incident frailty to account for potential differences in 25(OH)D concentrations associated with the timing of blood collection. Seasons were classified according to the calendar months of blood collection as spring (March–May), summer (June–August), autumn (September–November), and winter (December–February). In the CLHLS, blood samples were predominantly collected during spring and summer, with no samples collected during winter (Matchar et al. 2016), therefore, only the three observed seasonal categories were included in the corresponding models. In contrast, seasonal information could not be reliably derived for the HRS because the precise blood collection dates required for seasonal classification were unavailable in the public‐use dataset. Consequently, season of blood collection was not included as an adjustment variable in the HRS analyses. Seasonal distributions for the CLHLS and ELSA were shown in Supplementary Table S7.

### 2.5 Statistical Analysis

Baseline characteristics of the study population stratified by frailty status and 25(OH)D concentrations were presented in Supplementary Table S3. Continuous variables are expressed as means ± standard deviations (SD), and categorical variables are presented as counts (percentages). Between‐group comparisons were performed using the Wilcoxon rank‐sum test for continuous variables and the Pearson chi‐squared test or Fisher's exact test for categorical variables.

To evaluate the cross‐sectional association between baseline 25(OH)D concentrations and FI, multivariable linear regression models were fitted with the continuous FI score as the dependent variable. Serum 25(OH)D was analyzed both categorically and continuously. Participants were classified into low and normal vitamin D groups, with the normal group serving as the reference category; continuous analyses were conducted per 10 nmol/L increment in serum 25(OH)D. Three models were constructed: Model 1 was unadjusted; Model 2 was adjusted for age, sex, education, income, and marital status; and Model 3 was further adjusted for smoking status, drinking status, diabetes, hypertension, BMI, and physical activity. Results were presented as regression coefficients (*β*) with 95% confidence intervals (CI). Potential nonlinear associations between serum 25(OH)D concentrations and FI were further examined using restricted cubic splines.

To investigate the longitudinal association between baseline 25(OH)D concentrations and the risk of incident frailty, Cox proportional hazards regression models were used to estimate hazard ratios (HR) and 95% CIs among participants who were non‐frail at baseline. Serum 25(OH)D was analyzed both categorically and continuously, with continuous associations estimated per 10 nmol/L increment. The proportional hazards assumption was assessed using Schoenfeld residuals and Kaplan–Meier curves (Supplementary Figure S1), and no violation of this assumption was identified for the exposure variables. Restricted cubic splines were additionally used to assess potential nonlinear dose–response relationships between serum 25(OH)D concentrations and incident frailty risk.

In addition, subgroup analyses were conducted according to age, sex, BMI, diabetes, hypertension, and physical activity, and effect modification was evaluated by including multiplicative interaction terms in the models. Age subgroup cut‐offs were selected with reference to the decade‐based age stratification used in previous frailty research (Chamberlain et al. 2016), while considering the age distributions and subgroup sample sizes of the three cohorts (Vrettos et al. 2024). Specifically, the largest proportions of participants were observed in the 80–89 year age group in the CLHLS, the 60–69 year age group in the ELSA, and the 70–79 year age group in the HRS (Figure 1C–E). Accordingly, the lower boundaries of these age groups (80, 60, and 70 years, respectively) were selected as the cut‐offs to distinguish younger and older age strata within each cohort. This approach allowed us to examine potential effect modification by age in relation to each cohort's age structure, without assuming that the age strata defined by different cut‐offs were equivalent across cohorts. To address differences in age eligibility across cohorts, we additionally repeated the primary cross‐sectional and longitudinal analyses in the ELSA and HRS after restricting the study populations to participants aged ≥ 65 years, using the same analytical procedures and covariate adjustments as in the primary analyses. For subgroup analyses, BMI was dichotomised at 24 kg/m^2^ across all three cohorts as a harmonized analytical threshold to facilitate consistent cross‐cohort comparison; these categories were not intended to represent population‐specific clinical definitions of overweight. Because the CLHLS primarily comprises the oldest‐old and included relatively few participants with diabetes, diabetes stratification was not performed in the CLHLS subgroup analyses. Missing covariate data were handled using multiple imputation.

To assess the robustness of the findings, we conducted a series of sensitivity analyses. (1) We repeated the primary analyses after excluding participants with missing covariate data. (2) We restricted the analysis to participants who completed both follow‐up assessments to evaluate whether loss to follow‐up materially affected the results. (3) To minimize potential reverse causation, we performed a 2‐year lag analysis by excluding incident frailty cases occurring within the first 2 years of follow‐up; owing to the limited number of eligible participants and incident events remaining after applying this restriction in CLHLS, this analysis was conducted only in ELSA and HRS. (4) Because death may preclude the occurrence of frailty and therefore constitute a competing event, we additionally fitted competing‐risk models to account for the competing risks of death and incident frailty. Because mortality data after Wave 6 were unavailable in the ELSA due to privacy restrictions, this analysis was conducted only in the CLHLS and HRS. (5) We applied discrete‐time models as an alternative approach to the longitudinal analysis. (6) We used a commonly used FI cut‐off, with non‐frailty defined as FI < 0.21 and frailty as FI ≥ 0.21, and repeated the analyses accordingly (Kaskirbayeva et al. 2023; Qi et al. 2023).

All statistical analyses were performed using R software (version 4.4.1). All *p* values were two‐sided, and a *p* value < 0.05 was considered statistically significant.

## 3 Results

### 3.1 Baseline Characteristics of the Study Population

This study included 13,164 participants: 2038 from CLHLS (mean age, 85.42 years), 6061 from ELSA (mean age, 66.61 years), and 5065 from HRS (mean age, 74.55 years). Detailed baseline characteristics stratified by frailty status were presented in Table 1. In the CLHLS cohort, 379 participants were classified as frail at baseline. Compared with non‐frail participants, those with frailty were older, more often women, more likely to live in rural areas, and had lower education, lower physical activity levels, and lower serum vitamin D concentrations. In the ELSA cohort, frail participants showed a similar pattern, being older, more often women, less educated, less physically active, and having lower serum 25(OH)D concentrations than their non‐frail counterparts. Similar trends were observed in the HRS cohort. Baseline characteristics stratified by vitamin D status (normal and low) were provided in the Supplementary Materials Table S3.

**TABLE 1:** Baseline characteristics of participants for baseline frailty status.

Characteristic	CLHLS (*n* = 2038)			ELSA (*n* = 6061)			HRS (*n* = 5065)		
	No‐frailty *n* = 1659^a^	Frailty *n* = 379^a^	*p* ^b^	No‐frailty *n* = 4974^a^	Frailty *n* = 1087^a^	*p* ^b^	No‐frailty *n* = 3289^a^	Frailty *n* = 1776^a^	*p* ^b^
Age, years	83.00 ± 11.56	96.02 ± 8.33	< 0.001	65.76 ± 8.65	70.51 ± 9.96	< 0.001	73.92 ± 6.80	75.73 ± 7.53	< 0.001
Sex									
Female	810.0 (48.8%)	285.0 (75.2%)	< 0.001	2629.0 (52.9%)	693.0 (63.8%)	< 0.001	1821.0 (55.4%)	1123.0 (63.2%)	< 0.001
Male	849.0 (51.2%)	94.0 (24.8%)		2345.0 (47.1%)	394.0 (36.2%)		1468.0 (44.6%)	653.0 (36.8%)	
Residence/Birthplace^c^									
Urban	283.0 (17.1%)	64.0 (16.9%)	> 0.9	—	—	0.031	2220.0 (67.5%)	1144.0 (64.4%)	0.007
Rural	1376.0 (82.9%)	315.0 (83.1%)		—	—		856.0 (26.0%)	477.0 (26.9%)	
UK	—	—		4556.0 (91.6%)	1017.0 (93.6%)		—	—	
Out UK	—	—		418.0 (8.4%)	70.0 (6.4%)		—	—	
Missing	—	—		—	—		213.0 (6.5%)	155.0 (8.7%)	
Marital status									
Married or partnered	779.0 (47.0%)	55.0 (14.5%)	< 0.001	1288.0 (25.9%)	494.0 (45.4%)	< 0.001	1171.0 (35.6%)	833.0 (46.9%)	< 0.001
Not married	867.0 (52.3%)	324.0 (85.5%)		3685.0 (74.1%)	593.0 (54.6%)		2113.0 (64.2%)	943.0 (53.1%)	
Missing	13.0 (0.8%)	—		1.0 (0.0%)	—		5.0 (0.2%)	—	
Education levels^d^									
Low	938.0 (56.5%)	318.0 (83.9%)	< 0.001	1074.0 (21.6%)	467.0 (43.0%)	< 0.001	377.0 (11.5%)	475.0 (26.7%)	< 0.001
Middle	384.0 (23.1%)	41.0 (10.8%)		2495.0 (50.2%)	443.0 (40.8%)		1923.0 (58.5%)	1051.0 (59.2%)	
High	323.0 (19.5%)	14.0 (3.7%)		1052.0 (21.1%)	80.0 (7.4%)		987.0 (30.0%)	249.0 (14.0%)	
Missing	14.0 (0.8%)	6.0 (1.6%)		353.0 (7.1%)	97.0 (8.9%)		2.0 (0.1%)	1.0 (0.1%)	
Annual income/earnings, ￥/￡/$									
< 2500	241.0 (14.5%)	48.0 (12.7%)	0.11	3475.0 (69.9%)	1013.0 (93.2%)	< 0.001	2610.0 (79.4%)	1630.0 (91.8%)	< 0.001
2500–10000	585.0 (35.3%)	127.0 (33.5%)		456.0 (9.2%)	26.0 (2.4%)		154.0 (4.7%)	40.0 (2.3%)	
> 10000	756.0 (45.6%)	194.0 (51.2%)		1041.0 (20.9%)	45.0 (4.1%)		525.0 (16.0%)	106.0 (6.0%)	
Missing	77.0 (4.6%)	10.0 (2.6%)		2.0 (0.0%)	3.0 (0.3%)		—	—	
BMI, kg/m^2^									
< 18.5	359.0 (21.6%)	128.0 (33.8%)	< 0.001	39.0 (0.8%)	11.0 (1.0%)	< 0.001	54.0 (1.6%)	31.0 (1.7%)	< 0.001
18.5 ~ 25	1020.0 (61.5%)	172.0 (45.4%)		1435.0 (28.9%)	160.0 (14.7%)		986.0 (30.0%)	372.0 (20.9%)	
25 ~ 30	203.0 (12.2%)	14.0 (3.7%)		2168.0 (43.6%)	306.0 (28.2%)		1328.0 (40.4%)	596.0 (33.6%)	
≥ 30	51.0 (3.1%)	10.0 (2.6%)		1247.0 (25.1%)	493.0 (45.4%)		899.0 (27.3%)	758.0 (42.7%)	
Missing	26.0 (1.6%)	55.0 (14.5%)		85.0 (1.7%)	117.0 (10.8%)		22.0 (0.7%)	19.0 (1.1%)	
Smoking									
No	1319.0 (79.5%)	352.0 (92.9%)	< 0.001	1999.0 (40.2%)	315.0 (29.0%)	< 0.001	1509.0 (45.9%)	729.0 (41.0%)	0.004
Yes	329.0 (19.8%)	25.0 (6.6%)		2975.0 (59.8%)	771.0 (70.9%)		1757.0 (53.4%)	1033.0 (58.2%)	
Missing	11.0 (0.7%)	2.0 (0.5%)		—	1.0 (0.1%)		23.0 (0.7%)	14.0 (0.8%)	
Drinking						< 0.001			< 0.001
No	1359.0 (81.9%)	355.0 (93.7%)	< 0.001	425.0 (8.5%)	227.0 (20.9%)		1298.0 (39.5%)	1039.0 (58.5%)	
Yes	292.0 (17.6%)	22.0 (5.8%)		4192.0 (84.3%)	713.0 (65.6%)		1989.0 (60.5%)	735.0 (41.4%)	
Missing	8.0 (0.5%)	2.0 (0.5%)		357.0 (7.2%)	147.0 (13.5%)		2.0 (0.1%)	2.0 (0.1%)	
Hypertension									
No	1207.0 (72.8%)	251.0 (66.2%)	0.002	3227.0 (64.9%)	378.0 (34.8%)	< 0.001	1293.0 (39.3%)	288.0 (16.2%)	< 0.001
Yes	421.0 (25.4%)	126.0 (33.2%)		1747.0 (35.1%)	709.0 (65.2%)		1996.0 (60.7%)	1488.0 (83.8%)	
Missing	31.0 (1.9%)	2.0 (0.5%)		—	—		—	—	
Diabetes									
No	1616.0 (97.4%)	367.0 (96.8%)	0.7	4659.0 (93.7%)	849.0 (78.1%)	< 0.001	2608.0 (79.3%)	982.0 (55.3%)	< 0.001
Yes	36.0 (2.2%)	10.0 (2.6%)		315.0 (6.3%)	238.0 (21.9%)		681.0 (20.7%)	794.0 (44.7%)	
Missing	7.0 (0.4%)	2.0 (0.5%)		—	—		—	—	
Physical activity									
Active	297.0 (17.9%)	19.0 (5.0%)	< 0.001	4378.0 (88.0%)	453.0 (41.7%)	< 0.001	2567.0 (78.0%)	873.0 (49.2%)	< 0.001
Inactive	1305.0 (78.7%)	354.0 (93.4%)		595.0 (12.0%)	634.0 (58.3%)		713.0 (21.7%)	900.0 (50.7%)	
Missing	57.0 (3.4%)	6.0 (1.6%)		1.0 (0.0%)	—		9.0 (0.3%)	3.0 (0.2%)	
Serum 25(OH)D, nmol/L	44.60 ± 19.69	34.21 ± 16.37	< 0.001	49.78 ± 23.00	43.52 ± 24.68	< 0.001	81.86 ± 33.30	73.21 ± 35.9	< 0.001
Frailty index	0.13 ± 0.06	0.34 ± 0.08	< 0.001	0.09 ± 0.06	0.38 ± 0.11	< 0.001	0.13 ± 0.06	0.39 ± 0.12	< 0.001

Abbreviations: 25(OH)D: 25‐hydroxyvitamin D; BMI, body mass index; CLHLS, Chinese Longitudinal Healthy Longevity Survey; ELSA, English Longitudinal Study of Aging; HRS, Health and Retirement Study.

^a^ Mean (SD); *n* (%).

^b^ Wilcoxon rank sum test; Pearson's chi‐squared test; Fisher's exact test.

^c^ Residence/Birthplace includes urban/rural (CLHLS and HRS) and UK/Out UK (ELSA).

^d^ Education levels were harmonized into three categories (low, middle, high) across datasets. In CLHLS, education levels were defined by years of schooling: 0 years (low), 1–5 years (middle), and > 5 years (high). In ELSA and HRS, education levels were categorized as less than upper secondary (low), upper secondary and vocational training (middle), and tertiary (high). Income was expressed on an annual basis in local currency. In the CLHLS, it represented total household income in the previous year; in the ELSA, annualized individual employment earnings after tax; and in the HRS, respondent earnings in the previous calendar year.

### 3.2 Associations of Baseline 25(OH)D Concentrations With Frailty Index

At baseline, compared with normal 25(OH)D concentrations (> 50 nmol/L), low 25(OH)D concentrations (≤ 50 nmol/L) were significantly associated with higher FI scores across all three cohorts, and these associations remained statistically significant after multivariable adjustment (CLHLS: *β* = 0.013, 95% CI: 0.005–0.022; ELSA: *β* = 0.010, 95% CI: 0.004–0.015; HRS: *β* = 0.020, 95% CI: 0.011–0.029) (Table 2). When 25(OH)D was analyzed continuously, each 10 nmol/L increment in baseline 25(OH)D was associated with a 0.006 lower FI score in CLHLS (95% CI: −0.008 to −0.004), with corresponding differences of 0.003 lower in ELSA (95% CI: −0.004 to −0.001) and 0.002 lower in HRS (95% CI: −0.003 to −0.001) (Table 2). Consistent with these findings, low 25(OH)D concentrations were associated with higher odds of prevalent frailty across all three cohorts, whereas each 10 nmol/L increment in 25(OH)D was associated with lower odds of prevalent frailty (Table S4). Restricted cubic spline analyses further suggested a dose–response relationship between serum 25(OH)D concentrations and FI scores in all three cohorts (Figure 2A–C). In CLHLS, the association was nonlinear, with lower FI scores observed at higher 25(OH)D concentrations across the lower concentration range, followed by a plateau at higher concentrations (*p*_−overall_ < 0.001; *p*_−non‐linear_ < 0.001). Similar nonlinear associations were observed in ELSA and HRS (*p*_−overall_ < 0.01; *p*_−non‐linear_ < 0.01), where FI scores were lower at higher 25(OH)D concentrations across the lower concentration range, followed by a slight upward trend at higher concentrations.

**TABLE 2:** Cross‐sectional associations of baseline serum 25(OH)D concentrations with frailty index across the CLHLS, ELSA, and HRS.

Serum 25(OH)D	CLHLS	*p*	ELSA	*p*	HRS	*p*
	*β* (95% CI)		*β* (95% CI)		*β* (95% CI)	
Normal	ref		ref		ref	
Low	0.013 (0.005–0.022)	0.002	0.010 (0.004–0.015)	< 0.001	0.020 (0.011–0.029)	< 0.001
	—	—	—	—	—	—
Per 10 nmol/L	−0.006 (−0.008 to −0.004)	< 0.001	−0.003 (−0.004 to −0.001)	< 0.001	−0.002 (−0.003 to −0.001)	< 0.001

*Note:* Normal 25(OH)D concentrations were defined as > 50 nmol/L and low concentrations as ≤ 50 nmol/L. Models were adjusted for age, sex, education, marital status, income, body mass index, smoking, drinking, diabetes, hypertension, and physical activity; the CLHLS and ELSA were additionally adjusted for season.

Abbreviations: 25(OH)D, 25‐hydroxyvitamin D; CI, confidence interval; CLHLS, Chinese Longitudinal Healthy Longevity Survey; ELSA, English Longitudinal Study of Aging; HRS, Health and Retirement Study.

**FIGURE 2:**
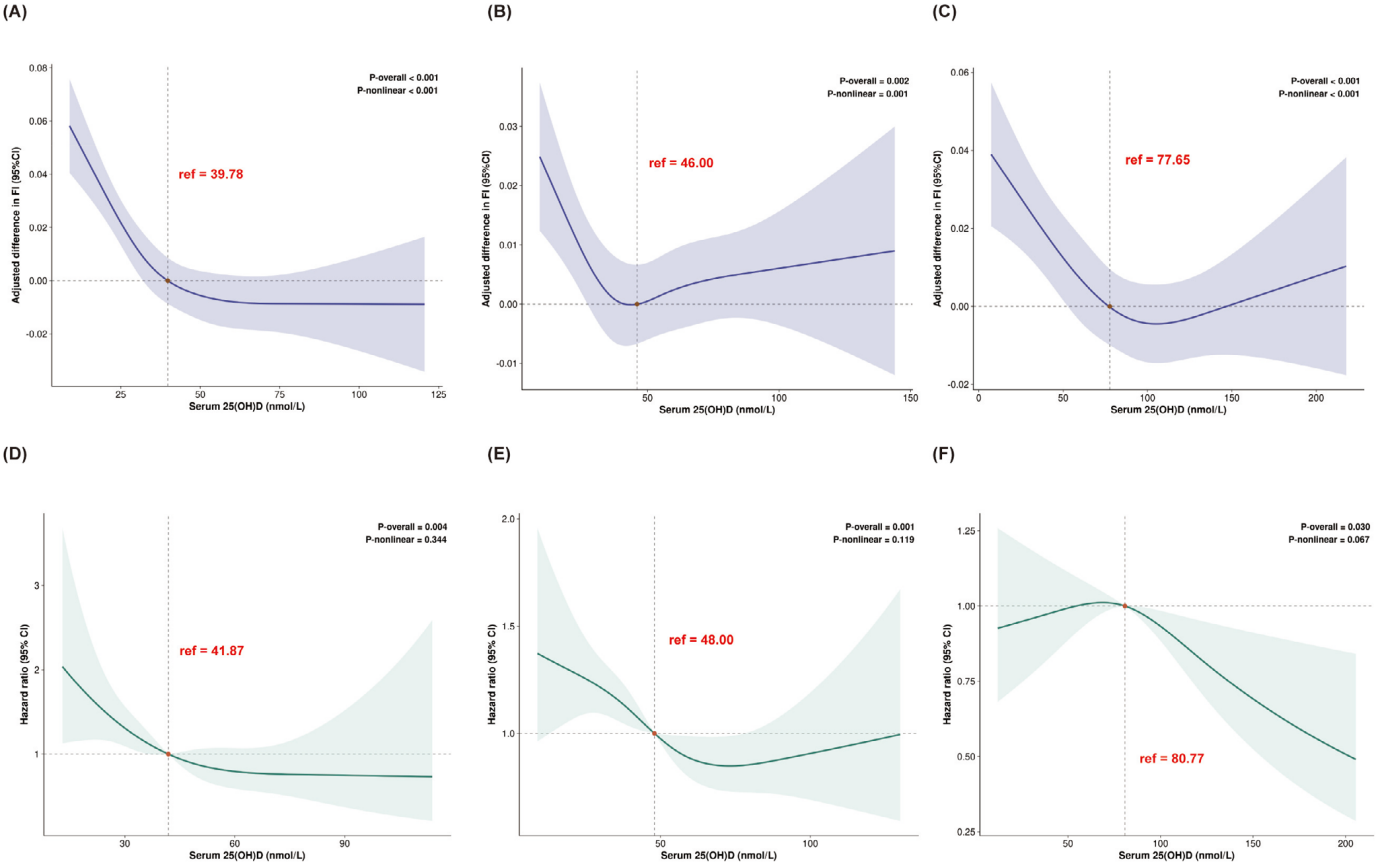
Restricted cubic spline analyses of the associations of 25(OH)D concentrations with FI and incident frailty. (A) Association between 25(OH)D concentrations and FI in CLHLS. (B) Association between 25(OH)D concentrations and FI in ELSA. (C) Association between 25(OH)D concentrations and FI in HRS. (D) Association between 25(OH)D concentrations and incident frailty in CLHLS. (E) Association between 25(OH)D concentrations and incident frailty in ELSA. (F) Association between 25(OH)D concentrations and incident frailty in HRS. Adjusted for age, sex, education, marital status, income, body mass index, smoking, drinking, diabetes, hypertension, and physical activity. CLHLS, Chinese Longitudinal Healthy Longevity Survey; ELSA, English Longitudinal Study of Aging; FI, frailty index; HRS, Health and Retirement Study.

### 3.3 Associations of Baseline 25(OH)D With Incident Frailty

Table 3 presented the associations between baseline serum 25(OH)D concentrations and the risk of incident frailty. After excluding participants who were frail at baseline and adjusting for age, sex, education, marital status, physical activity, and other covariates, low serum 25(OH)D concentrations were associated with a higher risk of incident frailty. Compared with participants with normal serum 25(OH)D concentrations, those with low 25(OH)D concentrations had a 76% higher risk of incident frailty in CLHLS (HR = 1.76, 95% CI: 1.19–2.60) and a 27% higher risk in ELSA (HR = 1.27, 95% CI: 1.08–1.50). A similar trend was observed in HRS (HR = 1.05, 95% CI: 0.86–1.27). When 25(OH)D was analyzed continuously, each 10 nmol/L increment in baseline 25(OH)D was associated with a lower risk of incident frailty in CLHLS, ELSA, and HRS, with corresponding HRs of 0.86 (95% CI: 0.77–0.95), 0.95 (95% CI: 0.92–0.99), and 0.97 (95% CI: 0.95–0.99), respectively. Further restricted cubic spline analysis did not identify a significant nonlinear dose–response relationship between baseline serum 25(OH)D concentrations and the risk of incident frailty (Figure 2D–F).

**TABLE 3:** Longitudinal associations of baseline serum 25(OH)D concentrations with incident frailty across the CLHLS, ELSA, and HRS.

Serum 25(OH)D	CLHLS	*p*	ELSA	*p*	HRS	*p*
	HR (95% CI)		HR (95% CI)		HR (95% CI)	
Normal	ref		ref		ref	
Low	1.76 (1.19–2.60)	0.004	1.27 (1.08–1.50)	0.004	1.05 (0.86–1.27)	0.624
	—	—	—	—	—	—
Per 10 nmol/L	0.86 (0.77–0.95)	0.003	0.95 (0.92–0.99)	0.013	0.97 (0.95–0.99)	0.005

*Note:* Longitudinal analyses were restricted to participants who were non‐frail at baseline. Normal 25(OH)D concentrations were defined as > 50 nmol/L and low concentrations as ≤ 50 nmol/L. Continuous associations were estimated per 10 nmol/L increment in baseline serum 25(OH)D. Models were adjusted for age, sex, education, marital status, income, body mass index, smoking, drinking, diabetes, hypertension, and physical activity; the CLHLS and ELSA were additionally adjusted for season.

Abbreviations: 25(OH)D, 25‐hydroxyvitamin D; CI, confidence interval; CLHLS, Chinese Longitudinal Healthy Longevity Survey; ELSA, English Longitudinal Study of Aging; HR, hazard ratio; HRS, Health and Retirement Study.

### 3.4 Subgroup Analyses

At baseline, subgroup analyses were conducted according to age, sex, BMI, hypertension, diabetes, and physical activity. In analyses of categorical 25(OH)D status, compared with participants with normal 25(OH)D concentrations (> 50 nmol/L), those with low 25(OH)D concentrations (≤ 50 nmol/L) tended to have higher FI scores across most subgroups (Supplementary Figure S2). In CLHLS, significant interactions were observed for age, BMI, and physical activity, whereas no significant interaction was detected in ELSA; in HRS, significant interactions were identified for sex, BMI, and physical activity.

Consistent results were observed when serum 25(OH)D was analyzed continuously, with each 10 nmol/L increment in baseline 25(OH)D generally associated with a lower FI (Supplementary Figure S3). In these analyses, age, sex, and physical activity significantly modified the association between 25(OH)D and FI in CLHLS; BMI and physical activity were significant effect modifiers in ELSA; and sex and physical activity were significant effect modifiers in HRS. Notably, physical activity showed a consistently significant interaction across all three cohorts, indicating its potential role as an important effect modifier in the association between vitamin D status and frailty.

Similarly, for the continuous association between baseline 25(OH)D and incident frailty, subgroup analyses were conducted according to age, sex, BMI, diabetes, hypertension, and physical activity (Supplementary Figure S4). Overall, the associations were broadly consistent across subgroups in all three cohorts, indicating that each 10 nmol/L increment in baseline 25(OH)D was associated with a lower risk of incident frailty. Notably, a significant interaction with physical activity was observed in all three cohorts, with this association being more pronounced among participants who were more physically active.

### 3.5 Sensitivity Analyses

To assess the robustness of our findings, we performed a series of sensitivity analyses. Overall, the results were consistent with those of the primary analyses, supporting the robustness of the observed associations. First, after excluding participants with missing covariate data in all three cohorts, we re‐examined the associations of baseline 25(OH)D concentrations with FI scores and incident frailty. After adjustment for the same set of covariates, the direction and statistical significance of the association between baseline 25(OH)D concentrations and FI scores remained largely unchanged, and the corresponding dose–response patterns were similar to those in the primary analyses (Supplementary Table S5; Figure S5). For the longitudinal complete‐case analysis, we applied the same baseline eligibility criterion as in the primary analysis, restricting the analysis to participants who were non‐frail at baseline and had complete covariate data. Each 10 nmol/L increment in serum 25(OH)D was associated with a lower risk of incident frailty in CLHLS (HR = 0.88, 95% CI: 0.80–0.98), ELSA (HR = 0.95, 95% CI: 0.92–0.99), and HRS (HR = 0.98, 95% CI: 0.95–1.00) (Figure 3). Consistent with the primary analysis, no clear dose–response relationship was observed. Second, to address differences in age eligibility across cohorts, we restricted the ELSA and HRS populations to participants aged ≥ 65 years. The cross‐sectional and longitudinal analyses yielded results largely consistent with those of the primary analyses (Supplementary Tables S8–S10). Third, when the analysis was restricted to participants who completed at least two follow‐up assessments, the results remained consistent across all three cohorts, further indicating that higher 25(OH)D concentrations were associated with a lower risk of incident frailty. Fourth, to minimize the potential influence of reverse causation, we conducted a 2‐year lag analysis in ELSA and HRS by excluding participants who developed incident frailty within the first 2 years of follow‐up; the results remained broadly unchanged. Fifth, when death was treated as a competing event for incident frailty, the competing‐risk models yielded results that were generally consistent with the primary analysis (CLHLS: HR = 0.90, 95% CI: 0.82–0.99; HRS: HR = 0.98, 95% CI: 0.96–1.00). Sixth, when a discrete‐time model was used as an alternative analytical approach, the results remained largely unchanged (CLHLS: HR = 0.86, 95% CI: 0.78–0.95; ELSA: HR = 0.93, 95% CI: 0.89–0.97; HRS: HR = 0.97, 95% CI: 0.94–0.99). Finally, when frailty was alternatively defined as FI ≥ 0.21, the direction of the associations remained generally consistent (CLHLS: HR = 0.94, 95% CI: 0.87–1.02; ELSA: HR = 0.92, 95% CI: 0.89–0.95; HRS: HR = 0.98, 95% CI: 0.95–1.00). Similarly, when 25(OH)D was analyzed categorically (low vs. normal) using the same approach as in the primary analysis, the results were consistent with the main findings (Supplementary Figure S6).

**FIGURE 3:**
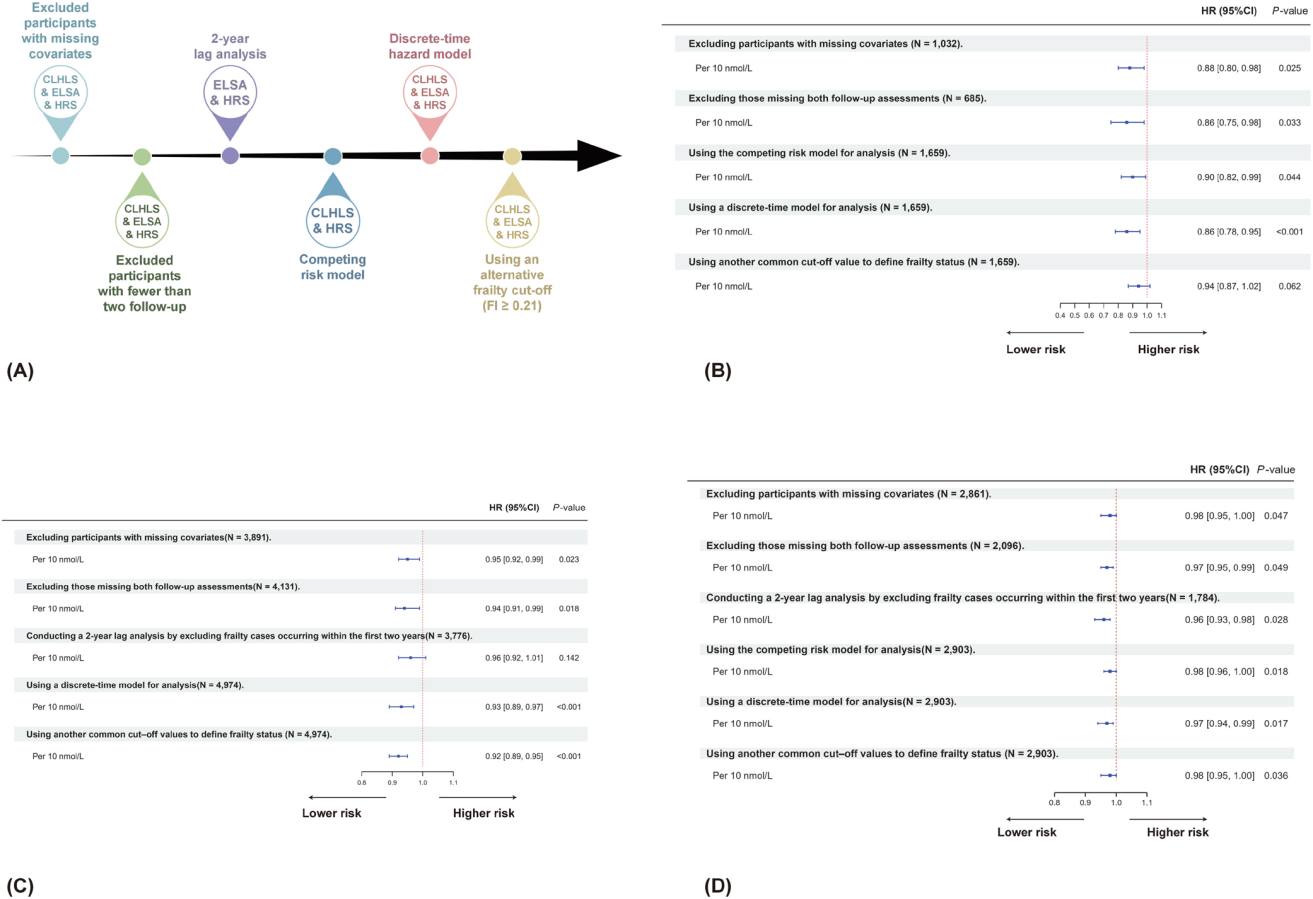
Sensitivity analyses of the longitudinal associations between baseline serum 25(OH)D concentrations and incident frailty (per 10 nmol/L increment) across the CLHLS, ELSA, and HRS. (A) Overview of the sensitivity analysis framework. (B) Sensitivity analysis results for CLHLS. (C) Sensitivity analysis results for ELSA. (D) Sensitivity analysis results for HRS. Sensitivity analyses included analyses restricted to participants with complete covariate data, analyses restricted to those with at least two follow‐up assessments, a 2‐year lag analysis, competing‐risk models, discrete‐time models, and analyses using an alternative FI cut‐off to define frailty. Adjusted for age, sex, education, marital status, income, body mass index, smoking, drinking, diabetes, hypertension, and physical activity. CI, confidence intervals; CLHLS, Chinese Longitudinal Healthy Longevity Survey; ELSA, English Longitudinal Study of Aging; FI, frailty index; HR, hazard ratios; HRS, Health and Retirement Study.

## 4 Discussion

This study used data from three prospective cohorts to examine the associations of baseline 25(OH)D concentrations with FI and incident frailty. The findings showed that, compared with participants with normal 25(OH)D concentrations, those with lower 25(OH)D concentrations had higher FI scores and a greater risk of incident frailty. Moreover, each 10 nmol/L increment in serum 25(OH)D was associated with lower FI and a lower risk of incident frailty. Additional analyses demonstrated a dose–response relationship between 25(OH)D concentrations and FI, suggesting that vitamin D may play a role in the development of frailty. Interestingly, for each 10 nmol/L increment in 25(OH)D, physical activity consistently modified its associations with both baseline FI and the risk of incident frailty across all three cohorts.

With advancing age, the physiological reserves required to maintain healthy aging progressively decline, and the contribution of nutritional factors to the development and progression of frailty has attracted increasing attention. Among these factors, vitamin D deficiency is common in middle‐aged and older adults and plays a central role in calcium–phosphate homeostasis and skeletal health. Consistent with our findings, a large cross‐sectional study by Zheng et al. reported an inverse association between serum 25(OH)D concentrations and frailty severity (Zheng et al. 2022). Similarly, a study by Gotaro et al. among nursing home residents found a close association between frailty and vitamin D deficiency (Kojima and Tanabe 2016). Importantly, the biological functions of vitamin D extend beyond skeletal health and include the maintenance of muscle mass and function, the regulation of inflammation, and the preservation of overall physiological homeostasis (Girgis et al. 2013). Frailty is a geriatric syndrome characterized by multisystem decline in physiological reserve and increased vulnerability to stressors, with key pathophysiological processes involving the neuromuscular, musculoskeletal, immune, and cardiovascular systems (Taylor et al. 2023). Therefore, low 25(OH)D concentrations may contribute to the accumulation of health deficits by accelerating declines in muscle function, impairing energy metabolism, and activating inflammatory pathways, thereby leading to higher FI scores and an increased risk of incident frailty. Previous studies have reported an association between low vitamin D status and sarcopenia, consistent with our recent findings from the HRS and ELSA (Zhang et al. 2026). Although the present study used the same baseline waves as our previous work, specifically HRS Wave 13 and ELSA Wave 6, differences in outcome‐specific eligibility criteria resulted in larger analytical samples in both cohorts, and we additionally included the CLHLS cohort. Furthermore, by prospectively assessing incident frailty during follow‐up, the present study extended beyond a cross‐sectional framework and provided evidence regarding the temporal association between baseline vitamin D status and subsequent frailty. This extension is clinically relevant because frailty reflects a broader decline in health and functional capacity than muscle impairment alone and is strongly associated with loss of independence and adverse health outcomes in later life (Clegg et al. 2013; Hoogendijk et al. 2019). The observed association with incident frailty therefore further highlights the potential importance of vitamin D as a modifiable nutritional factor in middle‐aged and older adults, particularly among individuals with low vitamin D status.

Interestingly, we observed a possible dose–response relationship between 25(OH)D concentrations and FI, and this pattern was broadly consistent across the three cohorts. Given that serum 25(OH)D concentrations vary seasonally (Lips et al. 2014), largely in response to changes in sunlight exposure, seasonal variation should also be considered when interpreting the observed dose–response patterns. Specifically, as serum 25(OH)D concentrations increased, FI scores showed a declining trend within a certain concentration range. However, beyond an apparent threshold, this decline gradually attenuated, particularly in the ELSA and HRS cohorts. The concentration at which this pattern changed appeared to vary across cohorts, suggesting potential population‐specific differences in the dose–response relationship. These findings suggest that the association between vitamin D and frailty may not be strictly linear, but instead may reflect an optimal range beyond which additional increases confer limited benefit. This observation is broadly consistent with previous studies. An analysis based on the UK Biobank reported a non‐linear, L‐shaped association between 25(OH)D concentrations and frailty risk (Zhang et al. 2024), and a recent systematic review and dose–response meta‐analysis likewise supported a dose–response relationship between serum 25(OH)D concentrations and frailty (Khakbaz et al. 2025). Together, this evidence suggests that the potential benefits of vitamin D may be concentrated primarily among individuals with lower baseline levels, whereas additional increases beyond a certain range may provide limited benefit. In the age‐stratified analyses, the associations between 25(OH)D concentrations and frailty appeared weaker in the older subgroups of the ELSA and HRS. This may partly reflect reduced sample sizes following stratification and greater interindividual heterogeneity among older adults in terms of health status, physiological function, and nutritional status. However, the interactions with age were not statistically significant, and these apparent differences should therefore be interpreted cautiously. The Endocrine Society has also noted that adults aged over 50 years with lower baseline 25(OH)D concentrations are more likely to benefit from vitamin D supplementation, but it does not recommend routine empirical supplementation above the dietary reference intake for the general healthy population. For adults aged 75 years and older, a cautious and individualized supplementation strategy is preferred (Demay et al. 2024). At the same time, randomized controlled trials (RCTs) have not supported additional health benefits from high‐dose daily vitamin D supplementation (Orkaby et al. 2022). Therefore, our findings further suggest that the focus of vitamin D intervention should not be the indiscriminate escalation of supplementation dose, but rather the identification of individuals with low 25(OH)D concentrations and the maintenance of 25(OH)D concentrations within an appropriate range. Further high‐quality RCTs are needed to clarify the optimal intervention threshold and target range of vitamin D for frailty prevention.

To our knowledge, this is the first study to use large‐scale, nationally representative cohort data from multiple countries to examine the population‐level association between vitamin D status and frailty risk. Across all three cohorts, higher baseline 25(OH)D concentrations were consistently associated with a lower risk of incident frailty. Moreover, age‐stratified analyses showed that, despite the use of different age cut‐offs in CLHLS, ELSA, and HRS (80, 60, and 70 years, respectively), the direction of the association overall remained unchanged, further supporting the consistency of the findings. Notably, the subgroups most likely to benefit from vitamin D supplementation may differ according to age structure, nutritional status, and physiological reserve, underscoring the importance of a precision nutrition approach (Aditya et al. 2025). In recent years, the evolving concept of precision nutrition has increasingly emphasized that nutritional interventions should be tailored to age, sex, lifestyle, and individual metabolic characteristics, rather than applied uniformly across populations (Guasch‐Ferré et al. 2025). Our findings further support the development of stratified intervention strategies based on age, vitamin D status, and lifestyle characteristics, rather than a single universal target. Both the cross‐cohort findings and the age‐stratified results provide epidemiological support for such individualized approaches. In addition, because frailty is not driven by a single risk factor but reflects the long‐term accumulation of nutritional deficiencies, reduced physical activity, chronic inflammation, and multisystem physiological decline (Taylor et al. 2023). Differences in continuous FI scores reflect variation in the overall burden of accumulated health deficits. Although the minimally important between‐person difference in FI may vary across populations and FI constructions, even relatively small increments in FI have been associated with subsequent adverse outcomes, including mortality (Thompson et al. 2021). We also examined the interaction between physical activity and 25(OH)D concentrations in relation to frailty outcomes. Previous studies have shown that vitamin D status and physical activity may jointly influence body composition and muscle function in older adults (Yang et al. 2020). Furthermore, a recent scoping review of RCTs suggested that combining exercise with vitamin D supplementation may provide additive benefits for outcomes such as Short Physical Performance Battery (SPPB) scores and stair‐climbing ability (Zhang et al. 2025). An important consideration is that physical activity itself is associated with vitamin D status, as more physically active individuals may spend more time outdoors and consequently receive greater sunlight exposure. Previous studies have reported higher serum 25(OH)D concentrations among individuals engaging in greater outdoor physical activity, and recent evidence suggests that this association may be largely related to time spent outdoors (Khan et al. 2024; Scragg and Camargo Jr 2008). Therefore, the observed interaction may partly reflect the close behavioral relationship between physical activity, outdoor exposure, and vitamin D status, rather than a purely biological synergistic effect. Further intervention studies are needed to clarify whether combining physical activity with vitamin D‐related strategies provides additional benefits for frailty prevention.

It is worth noting that, when vitamin D status was analyzed categorically at baseline, the interaction with physical activity for FI was not statistically significant in the ELSA cohort. This discrepancy may partly reflect the dynamic nature of serum 25(OH)D concentrations, which can be influenced by factors such as sunlight exposure, season, dietary intake, supplementation, and individual health characteristics (Lips et al. 2014). In addition, cut‐off values used to define low vitamin D status are not entirely consistent across studies and recommendations, and a single threshold may not fully capture differences in vitamin D status between individuals (Giustina et al. 2024). Because 25(OH)D was measured only at baseline in the present study, some participants may also have experienced changes in vitamin D status during follow‐up. Future studies incorporating repeated measurements of 25(OH)D may provide a more accurate assessment of vitamin D status and help clarify its interaction with physical activity.

This study has several limitations. First, as an observational study, it cannot completely exclude the possibility of residual confounding or reverse causation, despite adjustment for a range of potential confounders and the consistent direction of findings across multiple cohorts. Second, the specific vitamin D analytes measured and the assay platforms used differed across cohorts, which may have limited the direct comparability of absolute vitamin D concentrations across studies. Third, seasonal variation in serum 25(OH)D concentrations may have influenced the assessment of vitamin D status. Although season of blood collection was adjusted for in the CLHLS and ELSA, the seasonal distributions differed between these cohorts, with most CLHLS samples collected during summer and none during winter. Furthermore, seasonal adjustment could not be performed in the HRS because the necessary blood collection date information was unavailable in the public‐use dataset. These differences may have introduced residual seasonal influences and should be considered when interpreting and comparing findings across cohorts. Fourth, 25(OH)D concentrations were measured only at baseline and therefore did not capture changes in exposure over time; future studies incorporating repeated measurements would help to clarify the association between long‐term vitamin D exposure and the development and progression of frailty. Finally, although the same cumulative‐deficit framework was applied across all three cohorts, the specific FI indicators differed according to the measures available in each cohort. This should be considered when interpreting cross‐cohort comparisons, particularly absolute FI levels. Nevertheless, the cohort‐specific FI covered broadly comparable multidimensional health domains. Future studies should further develop and validate broadly applicable, cross‐culturally harmonized FI using conceptually comparable indicators while maintaining adequate multidomain coverage.

In conclusion, based on three large, nationally representative cohorts from China, England, and the United States, this study found that lower 25(OH)D concentrations were associated with higher FI and an increased risk of incident frailty. Further analyses suggested a possible non‐linear association between vitamin D and frailty, and indicated that physical activity may be an important effect modifier of this relationship. These findings suggest that vitamin D may represent a potentially modifiable factor in frailty prevention; however, its effects are likely to depend on an individual's baseline nutritional status and lifestyle context. Further prospective studies incorporating repeated measurements, together with RCTs, are needed to clarify the causal relationship between vitamin D and frailty and to inform frailty prevention strategies based on the synergistic integration of precision nutrition and physical activity.

## Author Contributions

**Mingkai Li:** conceptualization, methodology, writing – original draft, writing – review and editing, data curation, investigation, validation. **Yafei Zhao:** conceptualization, data curation, project administration. **Chenyang Li:** conceptualization, project administration. **Qinfei Guo:** conceptualization, project administration. **Chenhui Lv:** conceptualization, project administration. **Boqian Zhang:** conceptualization, project administration. **Lushan Xue:** conceptualization, project administration. **Cheng Zhang:** conceptualization, project administration. **Haifeng Zhao:** conceptualization, writing – review and editing, methodology, data curation, supervision, investigation, funding acquisition, writing – original draft.

## Funding

This study was supported by the National Natural Science Foundation of China (82574092), the Central Guiding Fund for the Development of Local Science and Technology (YDZJSX20231A056), the Research Project Supported by Shanxi Scholarship Council of China (2023‐103), and the Shanxi Province Higher Education “Billion Project” Science and Technology Guidance Project.

## Ethics Statement

This study was conducted in accordance with the Declaration of Helsinki. The CLHLS was approved by the Biomedical Ethics Committee of Peking University, China (IRB00001052–13074). The ELSA received ethical approval from the London Multi‐Centre Research Ethics Committee (MREC/01/2/91), and the HRS was approved by the University of Michigan Institutional Review Board and the National Institute on Aging (HUM00061128). Written informed consent was obtained from all participants in the three cohorts, and all studies were conducted in accordance with the relevant ethical guidelines and regulations.

## Conflicts of Interest

The authors declare no conflicts of interest.

## Supporting information


**Table S1:** The 40 items used to construct the frailty index in the CLHLS.
**Table S2:** The 28 items used to construct the frailty index in the ELSA and HRS.
**Table S3:** Baseline characteristics of participants for baseline serum 25(OH)D status.
**Table S4:** Cross‐sectional associations of serum 25(OH)D with prevalent frailty at baseline across three cohorts.
**Table S5:** Cross‐sectional associations of baseline serum 25(OH)D concentrations with frailty index across the CLHLS, ELSA, and HRS among participants with complete covariate data.
**Table S6:** Comparison of the components and operational definitions used to construct the frailty index across the CLHLS, ELSA, and HRS.
**Table S7:** Distribution of blood collection season across the CLHLS and ELSA cohorts.
**Table S8:** Cross‐sectional associations of baseline serum 25(OH)D concentrations with frailty index among adults aged ≥ 65 years in ELSA and HRS.
**Table S9:** Cross‐sectional associations of serum 25(OH)D with prevalent frailty at baseline among adults aged ≥ 65 years in ELSA and HRS.
**Table S10:** Longitudinal associations of baseline serum 25(OH)D concentrations with incident frailty among adults aged ≥ 65 years in ELSA and HRS.
**Figure S1:** Kaplan–Meier curves for incident frailty in participants with low and normal vitamin D status.
**Figure S2:** Cross‐sectional subgroup analyses of the associations between low versus normal baseline 25(OH)D concentrations and frailty index across the CLHLS, ELSA, and HRS.
**Figure S3:** Cross‐sectional subgroup analyses of the associations between baseline 25(OH)D concentrations (per 10 nmol/L increment) and frailty index across the CLHLS, ELSA, and HRS.
**Figure S4:** Longitudinal subgroup analyses of the associations between baseline 25(OH)D concentrations (per 10 nmol/L increment) and incident frailty across the CLHLS, ELSA, and HRS.
**Figure S5:** Restricted cubic spline analyses of the associations of 25(OH)D concentrations with FI and incident frailty (excluding participants with missing covariate data). (A) Association between 25(OH)D concentrations and FI in CLHLS. (B) Association between 25(OH)D concentrations and FI in ELSA. (C) Association between 25(OH)D concentrations and FI in HRS. (D) Association between 25(OH)D concentrations and incident frailty in CLHLS. (E) Association between 25(OH)D concentrations and incident frailty in ELSA. (F) Association between 25(OH)D concentrations and incident frailty in HRS. Adjusted for age, sex, education, marital status, income, body mass index, smoking, drinking, diabetes, hypertension, and physical activity, the CLHLS, and ELSA were additionally adjusted for season. CLHLS, Chinese Longitudinal Healthy Longevity Survey; ELSA, English Longitudinal Study of Aging; FI, frailty index; HRS, Health and Retirement Study.
**Figure S6:** Sensitivity analyses of the associations of 25(OH)D concentrations (normal/lower) with incident frailty in CLHLS, ELSA, and HRS. (A) Overview of the sensitivity analysis framework. (B) Sensitivity analysis results for CLHLS. (C) Sensitivity analysis results for ELSA. (D) Sensitivity analysis results for HRS. Sensitivity analyses included analyses restricted to participants with complete covariate data, analyses restricted to those with at least two follow‐up assessments, a 2‐year lag analysis, competing‐risk models, discrete‐time models, and analyses using an alternative FI cut‐off to define frailty. Adjusted for age, sex, education, marital status, income, body mass index, smoking, drinking, diabetes, hypertension, and physical activity, the CLHLS, and ELSA were additionally adjusted for season. CI, confidence intervals; CLHLS, Chinese Longitudinal Healthy Longevity Survey; ELSA, English Longitudinal Study of Aging; FI, frailty index; HR, hazard ratios; HRS, Health and Retirement Study.

**Supplementary S1.** Study designs of the CLHLS, ELSA, and HRS.

## Data Availability

The CLHLS dataset used in this study is available from the data management team through the Peking University Open Research Data platform upon submission of a data use agreement. The original data for the HRS and ELSA cohorts can be accessed by application through their respective official websites: HRS (https://hrs.isr.umich.edu/data‐products), ELSA (https://beta.ukdataservice.ac.uk/datacatalogue/series/series?id=200011). The CLHLS dataset can be accessed at the Peking University Open Research Data platform (https://opendata.pku.edu.cn/dataset.xhtml?persistentId=doi:10.18170/DVN/WBO7LK&version=2.0).
