# Supplementary material for: Association of Serum 25‐Hydroxyvitamin D With Frailty Across Three Prospective Cohorts: Effect Modification by Physical Activity
Source: Food Sci Nutr. 2026 Nov 13;14(11):e72589. doi: 10.1002/fsn3.72589 (PMC13765482; doi:10.1002/fsn3.72589)
Supplement: Supplementary file 1 [file FSN3-14-e72589-s001.docx]

**Supplemental Methods**

**Study designs of** **the CLHLS, ELSA, and HRS**

The Chinese Longitudinal Healthy Longevity Survey (CLHLS) was a prospective and national community-based longitudinal cohort study conducted in China. In baseline survey, a nationally representative sample of 9,093 participants aged ≥80 years was recruited from 23 provinces in mainland China in 1998. Subsequently, follow-up surveys and recruitment of new participants every 2–4 years (2000, 2002, 2005, 2008, 2011, and 2014) until 2018 were conducted. The 2002 wave extended its age range of the sampled elderly from 80 and over in the previous surveys to include the age range 65 to 79. In the most recent follow-up survey (2017–2018), 15,874 participants aged ≥65 years were interviewed, and mortality information for 2,226 individuals who died between 2014 and 2018 was obtained. The primary aim of this study was to investigate determinants of healthy longevity among older populations, with a focus on individuals aged ≥65 years. All participants underwent face-to-face interviews conducted by trained staff using standardized questionnaires (questionnaires administered to surviving participants and proxy respondents of deceased participants), to collect data on sociodemographic characteristics, socioeconomic status, lifestyle factors, mental health conditions, and so on. The survey collected detailed information on physical and cognitive function, including activities of daily living and cognitive status assessed by standardized instruments such as the Mini-Mental State Examination (MMSE). In addition, chronic diseases (including hypertension, diabetes, heart disease, and stroke) and anthropometric measurements indicators were recorded.^1^

The English Longitudinal Study of Ageing (ELSA) was a prospective cohort study conducted in England. In baseline survey, nationally representative sample of 11,391 participants aged ≥50 years was recruited in 2002-2003. For these participants, face-to-face interviews combined with self-completion questionnaires were biennially conducted by the trained staff to collect data on sociodemographic information, lifestyles, and health-related information, and further assess economic, physical, and mental health status. In wave 2 (2004-2005), which included 7,666 participants from the core members, nurse visit were added for the assessments of anthropometric (including height, weight, waist circumference, hip circumference, blood pressure, grip strength, and so on) and blood biochemical indicators (including total cholesterol, high-density lipoprotein cholesterol, low-density lipoprotein cholesterol, glycated hemoglobin, fasting blood glucose, and C-reactive protein). At the same time, the nurse visits were conducted every four years since wave 2.^2^

The Health and Retirement Study (HRS) was also a prospective and nationally representative cohort study of more than 37 000 individuals aged ≥50 in 23 000 households in the USA. For baseline survey, HRS recruited participants aged 51 to 61 years in 1992 combined with the Asset and Health Dynamics of the Oldest Old Study which enrolled participants aged ≥70 years in 1993. In 1998, HRS also merged two studies (the Children of the Depression and the War Babies) to make the sample fully representative of the USA population aged ≥50 years. The study, which has been fielded every 2 years since 1992, was established to provide a national resource for data on the changing health and economic circumstances associated with ageing at both individual and population levels. Participants were interviewed to collect data on sociodemographic information, lifestyles, and health-related information. Before 2004 (wave 7), most interviews were conducted by telephone, except for participants aged ≥80 who were offered face-to-face interviews. Since 2006 (wave 8), half of the participants performed enhanced face-to-face interviews (EFTF), while the other half carried out telephone interviews. Data collection has expanded to include biomarkers and genetics as well as much greater depth in psychology and social context. In the EFTF interviews, physical tests were conducted to collect data on height, weight, waist circumference, blood pressure, grip strength, and so on. Meanwhile, blood samples were assayed for five biochemical indicators: total cholesterol, high-density lipoprotein cholesterol, glycated hemoglobin, C-reactive protein, and cystatin C.^3^

**Vitamin D measurement**

In the CLHLS, Vitamin D measurements were derived from the 2012 biomarker substudy, which was conducted across eight longevity regions in China [4, 5]. Published methodological descriptions indicate that collected blood samples were placed in heparin-anticoagulated vacuum tubes and centrifuged at 2500 rpm at 20°C for 10 minutes; after plasma separation, the samples were initially frozen at −20°C, subsequently transported, and stored in the laboratory at −80°C. Vitamin D status was assessed using 25-hydroxyvitamin D3 [25(OH)D3], measured via an enzyme-linked immunoassay (ELISA) from Immunodiagnostic Systems (IDS, UK); published literature reports that its inter-batch and intra-batch coefficients of variation are <10% and <8%, respectively.

In ELSA, serum 25(OH)D was first measured at Wave 6 (2012–2013) [6]. According to data from the ELSA nurse visits, fasting blood samples were collected from participants during home visits, and samples for vitamin D analysis were obtained using plain red-top tubes. Blood collection for Wave 6 took place between January 2012 and July 2013. Laboratory analyses were performed at the Royal Victoria Infirmary in Newcastle upon Tyne, UK Serum 25(OH)D concentrations were measured using the DiaSorin Liaison 25-hydroxyvitamin D immunoassay This method is classified as a total 25(OH)D assay, indicating that it quantifies total circulating 25(OH)D rather than separately reporting 25(OH)D3 concentrations. Published literature has reported a detection limit of 3.0 nmol/L. All samples were analysed in duplicate, and the overall intra- and inter-batch coefficients of variation ranged from 8.7% to 9.4% .

In HRS, vitamin D data were obtained from the 2016 Venous Blood Study (VBS) [7,8]. Venous blood samples were collected by trained phlebotomists during home visits; fasting was recommended but not required, and each participant’s fasting status was recorded [7]. Blood was collected into several types of tubes, including three serum separator tubes (SSTs). After on-site centrifugation, the SSTs were shipped overnight to a CLIA-certified laboratory at the University of Minnesota and processed within 24 hours of arrival, corresponding to within 48 hours of blood collection [7]. Vitamin D concentrations were measured using liquid chromatography–tandem mass spectrometry (LC–MS/MS).

**Reference**

1. Center for Healthy Aging and Development Studies. The Chinese Longitudinal Healthy Longevity Survey (CLHLS)-Longitudinal Data (1998–2018). Version 2. Peking University Open Research Data Platform. 2020. doi:https://doi.org/10.18170/DVN/WBO7LK.
2. Steptoe A, Breeze E, Banks J, Nazroo J. Cohort profile: the English longitudinal study of ageing. Int J Epidemiol. 2013;**42**(6):1640-1648. doi: https://doi.org/10.1093/ije/dys168
3. Sonnega A, Faul JD, Ofstedal MB, Langa KM, Phillips JW, Weir DR. Cohort Profile: the Health and Retirement Study (HRS). Int J Epidemiol. 2014;**43**(2):576-585. doi: https://doi.org/10.1093/ije/dyu067
4. Matchar DB, Chei CL, Yin ZX, et al. Vitamin D Levels and the Risk of Cognitive Decline in Chinese Elderly People: the Chinese Longitudinal Healthy Longevity Survey. J Gerontol A Biol Sci Med Sci. 2016;71(10):1363–1368. doi: https://doi.org/10.1093/gerona/glw128
5. Zhu A, Zeng Y, Ji JS. Residential Greenness Alters Serum 25(OH)D Concentrations: A Longitudinal Cohort of Chinese Older Adults. J Am Med Dir Assoc. 2020;21(12):1968–1972.e2. doi: https://doi.org/10.1016/j.jamda.2020.04.026
6. Aspell N, Laird E, Healy M, et al. The Prevalence and Determinants of Vitamin D Status in Community-Dwelling Older Adults: Results from the English Longitudinal Study of Ageing (ELSA). Nutrients. 2019;11(6):1253. doi:https://doi.org/10.3390/nu11061253
7. Crimmins E, Faul J, Thyagarajan B, Weir D. Venous blood collection and assay protocol in the 2016 Health and Retirement Study (2016 Venous Blood Study, VBS): Documentation Report. University of Michigan; 2017
8. Crimmins EM, Faul J, Kim JK, Thyagarajan B, Weir D. VBS 2016 Supplemental File: Cytokines, Vitamin D, and IGF 1 from the 2016 Venous Blood Study. University of Michigan; 2020
