# Supplementary material for: Association of Serum 25‐Hydroxyvitamin D With Frailty Across Three Prospective Cohorts: Effect Modification by Physical Activity
Source: Food Sci Nutr. 2026 Nov 13;14(11):e72589. doi: 10.1002/fsn3.72589 (PMC13765482; doi:10.1002/fsn3.72589)
Supplement: Supplementary file 2 [file FSN3-14-e72589-s002.docx]

**Additional file : Table S1-S8 and Figures S1-S6**

**Table S1. The 40 items used to construct the frailty index in the CLHLS**

**Table S2. The 28 items used to construct the frailty index in the ELSA and HRS**

**Table S3 Baseline characteristics of participants for baseline Serum 25(OH)D status**

**Table S4 Cross-sectional associations of serum 25(OH)D with prevalent frailty at baseline across three cohorts**

**Table S5 Cross-sectional associations of baseline serum 25(OH)D concentrations with frailty index across the CLHLS, ELSA, and HRS among participants with complete covariate data**

**Table S6. Comparison of the components and operational definitions used to construct the frailty index across the CLHLS, ELSA, and HRS**

**Table S7. Distribution of blood collection season across the CLHLS and ELSA cohorts**

**Table S8. Cross-sectional associations of baseline serum 25(OH)D concentrations with frailty index among adults aged ≥65 years in ELSA and HRS**

**Table S9. Cross-sectional associations of serum 25(OH)D with prevalent frailty at baseline among adults aged ≥65 years in ELSA and HRS**

**Table S10. Longitudinal associations of baseline serum 25(OH)D concentrations with incident frailty among adults aged ≥65 years in ELSA and HRS**

**Figure S1 Kaplan–Meier curves for incident frailty in participants with low and normal vitamin D status**

**Figure S2. Cross-sectional subgroup analyses of the associations between low versus normal baseline 25(OH)D concentrations and frailty index across the CLHLS, ELSA, and HRS.**

**Figure S3. Cross-sectional subgroup analyses of the associations between baseline 25(OH)D concentrations (per 10 nmol/L increment) and frailty index across the CLHLS, ELSA, and HRS.**

**Figure S4. Longitudinal subgroup analyses of the associations between baseline 25(OH)D concentrations (per 10 nmol/L increment) and incident frailty across the CLHLS, ELSA, and HRS.**

**Figure S5 Restricted cubic spline analyses of the associations of 25(OH)D concentrations with FI and incident frailty**

**Figure S6 Sensitivity analyses of the associations of 25(OH)D concentrations (Normal/lower) with incident frailty in CLHLS, ELSA and HRS.**

**Table S1. The 40 items used to construct the frailty index in the CLHLS**

|  | **Variables** | **Cut-off value** |
| --- | --- | --- |
| 1 | Self‐reported health | Very good = 0; good = 0.25; okay= 0.5; bad = 0.75; very bad = 1 |
| 2 | Feel fearful or anxious | Always = 1; often = 0.75; sometimes = 0.5; seldom = 0.25; rarely or never = 0 |
| 3 | Feel useless with age | Always = 1; often = 0.75; sometimes = 0.5; seldom = 0.25; rarely or never = 0 |
| 4 | Bathing | Without assistance = 0; one part assistance = 0.5; more than one part assistance = 1 |
| 5 | Dressing | Without assistance = 0; one part assistance = 0.5; more than one part assistance = 1 |
| 6 | Toileting | Without assistance = 0; one part assistance = 0.5; more than one part assistance = 1 |
| 7 | Transferring | Without assistance = 0; one part assistance = 0.5; more than one part assistance = 1 |
| 8 | Continence | Without assistance = 0; one part assistance = 0.5; more than one part assistance = 1 |
| 9 | Feeding | Without assistance = 0; one part assistance = 0.5; more than one part assistance = 1 |
| 10 | Visual function | Can see and distinguish = 0; can see only = 0.5; can’t see = 1; blind = 1 |
| 11 | Rhythm of heart | ≥ 80bpm = 1; <80bpm = 0 |
| 12 | Hand behind neck | Both hands = 0; left hand = 0.5; right hand = 0.5; neither hand = 1 |
| 13 | Hand behind lower back | Both hands = 0; left hand = 0.5; right hand = 0.5; neither hand = 1 |
| 14 | Able to stand up from sitting | Yes, without using hands = 0; Yes, using hands = 0.5; No = 1 |
| 15 | Able to pick up a book from the floor | Yes, standing = 0; Yes, sitting = 0.5; No = 1 |
| 16 | Able to carry 5kg weight | Yes = 0; a little difficult = 0.5; No = 1 |
| 17 | Able to walk a distance of 1 kilometer | Yes = 0; a little difficult = 0.5; No = 1 |
| 18 | Number of times suffering from serious illness in the past two years | Yes = 1; No = 0 |
| 19 | Hypertension | Yes = 1; No = 0 |
| 20 | Diabetes | Yes = 1; No = 0 |
| 21 | Heart disease | Yes = 1; No = 0 |
| 22 | Stroke or CVD | Yes = 1; No = 0 |
| 23 | Bronchitis, emphysema, pneumonia, or asthma | Yes = 1; No = 0 |
| 24 | Tuberculosis | Yes = 1; No = 0 |
| 25 | Cancer | Yes = 1; No = 0 |
| 26 | Gastric or duodenal ulcer | Yes = 1; No = 0 |
| 27 | Parkinson | Yes = 1; No = 0 |
| 28 | Bedsore | Yes = 1; No = 0 |
| 29 | Able to hear | Yes = 0; with or partly with hearing aid = 0.5; No = 1 |
| 30 | Interviewer rated health | Surprisingly healthy = 0; relatively healthy = 0; moderately ill = 0.5; very ill = 1 |
| 31 | Look on the bright side of things | Always = 0; often = 0.25; sometimes = 0.5; seldom = 0.75; rarely or never = 1 |
| 32 | Keep my belongings neat and clean | Always = 0; often = 0.25; sometimes = 0.5; seldom = 0.75; rarely or never = 1 |
| 33 | Make own decisions | Always = 0; often = 0.25; sometimes = 0.5; seldom = 0.75; rarely or never = 1 |
| 34 | Housework at present | Almost every day = 0; not daily, but once for a week = 0.25; not weekly, but at least once for a month = 0.5; not monthly, but sometimes = 0.75; never = 1 |
| 35 | Able to use chopsticks to eat | Yes = 0; No = 1 |
| 36 | Number of steps used to turn around a 360 degree turn without help | ≥ 6 steps = 1; <6 steps = 0 |
| 37 | Cataract | Yes = 1; No = 0 |
| 38 | Glaucoma | Yes = 1; No = 0 |
| 39 | Prostate Tumor | Yes = 1; No = 0 |
| 40 | Dementia | Yes = 1; No = 0 |

**Table S2. The 28 items used to construct the frailty index in the ELSA and HRS**

|  | **Variables** | **Cut-off value** |
| --- | --- | --- |
| 1 | Self-reported physician diagnosed hypertension | Yes = 1, No = 0 |
| 2 | Self-reported physician diagnosed diabetes | Yes = 1, No = 0 |
| 3 | Self-reported physician diagnosed cancer | Yes = 1, No = 0 |
| 4 | Self-reported physician diagnosed arthritis | Yes = 1, No = 0 |
| 5 | Self-reported physician diagnosed chronic lung disease | Yes = 1, No = 0 |
| 6 | Self-reported physician diagnosed any emotional, nervous, or psychiatric problems | Yes = 1, No = 0 |
| 7 | Self-reported physician diagnosed memory-related disease**^1^** | Yes = 1, No = 0 |
| 8 | Self-reported eyesight (while using lenses if appropriate) | Poor or fair = 1, excellent, very good, or good = 0 |
| 9 | Self-reported hearing (while using hearing aid  if appropriate) | Poor or fair = 1, excellent, very good, or good = 0 |
| 10 | Self-reported general health status | Very poor or poor = 1, Very good, good, or fair = 0 |
| 11 | Difficulty with dressing | Yes = 1, No = 0 |
| 12 | Difficulty with bathing or showering | Yes = 1, No = 0 |
| 13 | Difficulty with eating | Yes = 1, No = 0 |
| 14 | Difficulty with getting in and out of bed | Yes = 1, No = 0 |
| 15 | Difficulty with using the toilet | Yes = 1, No = 0 |
| 16 | Difficulty with managing money | Yes = 1, No = 0 |
| 17 | Difficulty with taking medication | Yes = 1, No = 0 |
| 18 | Difficulty with shopping for groceries | Yes = 1, No = 0 |
| 19 | Difficulty with preparing meals | Yes = 1, No = 0 |
| 20 | Mobility: difficulty with walking 100 yards or one block | Yes = 1, No = 0 |
| 21 | Mobility: difficulty with getting up from a chair after sitting for long periods | Yes = 1, No = 0 |
| 22 | Mobility: difficulty with climbing several flights of stairs without resting | Yes = 1, No = 0 |
| 23 | Mobility: difficulty with lifting or carrying weights over 10 pounds | Yes = 1, No = 0 |
| 24 | Mobility: difficulty with picking up a coin from the table | Yes = 1, No = 0 |
| 25 | Mobility: difficulty with stooping, kneeling, or crouching | Yes = 1, No = 0 |
| 26 | Mobility: difficulty with reaching arms above shoulder level | Yes = 1, No = 0 |
| 27 | Depression**^2^**: CESD-8 questionnaire | CESD-8 ≥ 4 = 1, <4 = 0 |
| 28 | Cognition**^3^**: (memory test score + orientation test score) / 14 | Continuous variable, ranging from 0 to 1 |
| **^1^**Memory-related disease included Alzheimer’s disease, dementia, organic brain senility, and other serious memory impairment. | | |
| **^2^**Depression was assessed by the Center for Epidemiologic Studies Depression Scale (CESD). In the ELSA and HRS, CESD-8 was used, and the total score ranged from 0 to 8. A higher score indicated more severe depressive symptoms. | | |
| **^3^**The memory score was the average of words which were not recalled in the immediate and delayed word recall tasks. The memory score ranged from 0 to 10. The orientation test comprised 4 questions about the day of the week, the month, the date of the month, and the year. One point was given for each wrong answer, and the range was from 0 to 4. | | |

**Table S3 Baseline characteristics of participants for baseline Serum 25(OH)D status**

| Characteristic | CLHLS(n = 2038) | | | ELSA(n = 6061) | | | HRS(n = 5065) | | |
| --- | --- | --- | --- | --- | --- | --- | --- | --- | --- |
|  | Normal  n = 634^1^ | Deficient  n = 1404^1^ | p-value^2^ | Normal  n = 2606^1^ | Deficient  n = 3455^1^ | p-value^2^ | Normal  n = 4063^1^ | Deficient  n = 1002^1^ | p-value^2^ |
| Age, years | 82.08 ± 11.67 | 86.93 ± 12.04 | <0.001 | 66.53 ± 8.62 | 66.67 ± 9.42 | 0.6 | 74.75 ± 7.06 | 73.78 ± 7.26 | <0.001 |
| Sex |  |  | <0.001 |  |  | 0.14 |  |  | 0.5 |
| Female | 239.0 (37.7%) | 856.0 (61.0%) |  | 1400.0 (53.7%) | 1922.0 (55.6%) |  | 2372.0 (58.4%) | 572.0 (57.1%) |  |
| Male | 395.0 (62.3%) | 548.0 (39.0%) |  | 1206.0 (46.3%) | 1533.0 (44.4%) |  | 1691.0 (41.6%) | 430.0 (42.9%) |  |
| Residence/Birthplace^3^ |  |  | <0.001 |  |  | 0.001 |  |  | <0.001 |
| Urban | 78.0 (12.3%) | 269.0 (19.2%) |  | - | - |  | 2728.0 (67.1%) | 636.0 (63.5%) |  |
| Rural | 556.0 (87.7%) | 1135.0 (80.8%) |  | - | - |  | 1072.0 (26.4%) | 261.0 (26.0%) |  |
| UK | - | - |  | 2430.0 (93.2%) | 3143.0 (91.0%) |  | - | - |  |
| Out UK | - | - |  | 176.0 (6.8%) | 312.0 (9.0%) |  | - | - |  |
| Missing | - | - |  | - | - |  | 263.0 (6.5%) | 105.0 (10.5%) | <0.001 |
| Marital status |  |  | <0.001 |  |  | <0.001 |  |  |  |
| Married or partnered | 337.0 (53.2%) | 497.0 (35.4%) |  | 630.0 (24.2%) | 1152.0 (33.3%) |  | 1528.0 (37.6%) | 476.0 (47.5%) |  |
| Not married | 292.0 (46.1%) | 899.0 (64.0%) |  | 1975.0 (75.8%) | 2303.0 (66.7%) |  | 2530.0 (62.3%) | 526.0 (52.5%) |  |
| Missing | 5.0 (0.8%) | 8.0 (0.6%) |  | - | - |  | 5.0 (0.1%) | - |  |
| Education levels^4^ |  |  | <0.001 |  |  | 0.034 |  |  | <0.001 |
| Low | 313.0 (49.4%) | 943.0 (67.2%) |  | 614.0 (23.6%) | 927.0 (26.8%) |  | 593.0 (14.6%) | 259.0 (25.8%) |  |
| Middle | 162.0 (25.6%) | 263.0 (18.7%) |  | 1298.0 (49.8%) | 1640.0 (47.5%) |  | 2389.0 (58.8%) | 585.0 (58.4%) |  |
| High | 157.0 (24.8%) | 180.0 (12.8%) |  | 501.0 (19.2%) | 631.0 (18.3%) |  | 1079.0 (26.6%) | 157.0 (15.7%) |  |
| Missing | 2.0 (0.3%) | 18.0 (1.3%) |  | 193.0 (7.4%) | 257.0 (7.4%) |  | - | 1.0 (0.1%) |  |
| Annual income/earnings, ￥/￡/$ |  |  | 0.032 |  |  | 0.020 |  |  | 0.5 |
| <2500 | 108.0 (17.0%) | 181.0 (12.9%) |  | 1976.0 (75.8%) | 2512.0 (72.7%) |  | 3412.0 (84.0%) | 828.0 (82.6%) |  |
| 2500-10000 | 210.0 (33.1%) | 502.0 (35.8%) |  | 205.0 (7.9%) | 277.0 (8.0%) |  | 151.0 (3.7%) | 43.0 (4.3%) |  |
| >10000 | 296.0 (46.7%) | 654.0 (46.6%) |  | 423.0 (16.2%) | 663.0 (19.2%) |  | 500.0 (12.3%) | 131.0 (13.1%) |  |
| Missing | 20.0 (3.2%) | 67.0 (4.8%) |  | 2.0 (0.1%) | 3.0 (0.1%) |  | - | - |  |
| BMI, kg/m^2^ |  |  | 0.7 |  |  | <0.001 |  |  | <0.001 |
| <18.5 | 148.0 (23.3%) | 339.0 (24.1%) |  | 26.0 (1.0%) | 24.0 (0.7%) |  | 59.0 (1.5%) | 26.0 (2.6%) |  |
| 18.5 ~ 25 | 374.0 (59.0%) | 818.0 (58.3%) |  | 798.0 (30.6%) | 797.0 (23.1%) |  | 1154.0 (28.4%) | 204.0 (20.4%) |  |
| 25 ~ 30 | 74.0 (11.7%) | 143.0 (10.2%) |  | 1132.0 (43.4%) | 1342.0 (38.8%) |  | 1583.0 (39.0%) | 341.0 (34.0%) |  |
| ≥30 | 16.0 (2.5%) | 45.0 (3.2%) |  | 575.0 (22.1%) | 1165.0 (33.7%) |  | 1237.0 (30.4%) | 420.0 (41.9%) |  |
| Missing | 22.0 (3.5%) | 59.0 (4.2%) |  | 75.0 (2.9%) | 127.0 (3.7%) |  | 30.0 (0.7%) | 11.0 (1.1%) |  |
| Smoking |  |  | <0.001 |  |  | 0.2 |  |  | <0.001 |
| No | 476.0 (75.1%) | 1195.0 (85.1%) |  | 1019.0 (39.1%) | 1295.0 (37.5%) |  | 1842.0 (45.3%) | 396.0 (39.5%) |  |
| Yes | 152.0 (24.0%) | 202.0 (14.4%) |  | 1586.0 (60.9%) | 2160.0 (62.5%) |  | 2186.0 (53.8%) | 604.0 (60.3%) |  |
| Missing | 6.0 (0.9%) | 7.0 (0.5%) |  | - | - |  | 35.0 (0.9%) | 2.0 (0.2%) |  |
| Drinking |  |  | <0.001 |  |  | <0.001 |  |  | <0.001 |
| No | 493.0 (77.8%) | 1221.0 (87.0%) |  | 218.0 (8.4%) | 434.0 (12.6%) |  | 1782.0 (43.9%) | 555.0 (55.4%) |  |
| Yes | 136.0 (21.5%) | 178.0 (12.7%) |  | 2238.0 (85.9%) | 2667.0 (77.2%) |  | 2278.0 (56.1%) | 446.0 (44.5%) |  |
| Missing | 5.0 (0.8%) | 5.0 (0.4%) |  | 150.0 (5.8%) | 354.0 (10.2%) |  | 3.0 (0.1%) | 1.0 (0.1%) |  |
| Hypertension |  |  | 0.5 |  |  | <0.001 |  |  | <0.001 |
| No | 464.0 (73.2%) | 994.0 (70.8%) |  | 1623.0 (62.3%) | 1982.0 (57.4%) |  | 1332.0 (32.8%) | 249.0 (24.9%) |  |
| Yes | 159.0 (25.1%) | 388.0 (27.6%) |  | 983.0 (37.7%) | 1473.0 (42.6%) |  | 2731.0 (67.2%) | 753.0 (75.1%) |  |
| Missing | 11.0 (1.7%) | 22.0 (1.6%) |  | - | - |  | - | - |  |
| Diabetes |  |  | 0.024 |  |  | <0.001 |  |  | <0.001 |
| No | 625.0 (98.6%) | 1358.0 (96.7%) |  | 2417.0 (92.7%) | 3091.0 (89.5%) |  | 2938.0 (72.3%) | 652.0 (65.1%) |  |
| Yes | 9.0 (1.4%) | 37.0 (2.6%) |  | 189.0 (7.3%) | 364.0 (10.5%) |  | 1125.0 (27.7%) | 350.0 (34.9%) |  |
| Missing | - | 9.0 (0.6%) |  | - | - |  | - | - |  |
| Physical activity |  |  | 0.023 |  |  | <0.001 |  |  | <0.001 |
| Inactive | 119.0 (18.8%) | 197.0 (14.0%) |  | 395.0 (15.2%) | 834.0 (24.1%) |  | 1190.0 (29.3%) | 423.0 (42.2%) |  |
| Active | 495.0 (78.1%) | 1164.0 (82.9%) |  | 2211.0 (84.8%) | 2620.0 (75.8%) |  | 2862.0 (70.4%) | 578.0 (57.7%) |  |
| Missing | 20.0 (3.2%) | 43.0 (3.1%) |  | - | - |  | 11.0 (0.3%) | 1.0 (0.1%) |  |
| Serum 25(OH)D, nmol/L | 65.44 ± 15.99 | 32.38 ± 9.93 | <0.001 | 70.41 ± 17.02 | 32.25 ± 10.89 | <0.001 | 90.15 ± 28.34 | 32.94 ± 11.36 | <0.001 |
| Frailty index | 0.14 ± 0.08 | 0.19 ± 0.11 | <0.001 | 0.12 ± 0.12 | 0.15 ± 0.14 | <0.001 | 0.21 ± 0.1 | 0.27 ± 0.17 | <0.001 |
| ^1^Mean (SD); n (%) | | | | | | | | | |
| ^2^Wilcoxon rank sum test; Pearson's Chi-squared test; Fisher's exact test | | | | | | | | | |
| ^3^Residence/Birthplace includes urban/rural (CLHLS and HRS) and UK/Out UK (ELSA) | | | | | | | | | |
| ^4^Education levels were harmonized into three categories (Low, Middle, High) across datasets. In CLHLS, education levels were defined by years of schooling: 0 years (Low), 1–5 years (Middle), and >5 years (High). In ELSA and HRS, education levels were categorized as less than upper secondary (Low), upper secondary and vocational training (Middle), and tertiary (High). Income was expressed on an annual basis in local currency. In the CLHLS, it represented total household income in the previous year; in the ELSA, annualised individual employment earnings after tax; and in the HRS, respondent earnings in the previous calendar year. Abbreviation: CLHLS: Chinese Longitudinal Healthy Longevity Survey; ELSA: English Longitudinal Study of Ageing; HRS: Health and Retirement Study; BMI: body mass index; 25(OH)D: 25-hydroxyvitamin D. | | | | | | | | | |

**Figure S1 Kaplan–Meier curves for incident frailty in participants with low and normal vitamin D status**

(A) CLHLS; (B) ELSA; (C) HRS.

**Figure S2. Cross-sectional subgroup analyses of the associations between low versus normal baseline 25(OH)D concentrations and frailty index across the CLHLS, ELSA, and HRS.**

(A)CLHLS; (B) ELSA; (C) HRS.

**Figure S3. Cross-sectional subgroup analyses of the associations between baseline 25(OH)D concentrations (per 10 nmol/L increment) and frailty index across the CLHLS, ELSA, and HRS.**

(A) CLHLS; (B) ELSA; (C) HRS.

**Figure S4. Longitudinal subgroup analyses of the associations between baseline 25(OH)D concentrations (per 10 nmol/L increment) and incident frailty across the CLHLS, ELSA, and HRS.**

(A) CLHLS; (B) ELSA; (C) HRS.

**Table S4 Cross-sectional associations of serum 25(OH)D with prevalent frailty at baseline across three cohorts**

| **Serum 25(OH)D** | **CLHLS, OR (95% CI)** | ***P*** | **ELSA, OR (95% CI)** | ***P*** | **HRS, OR (95% CI)** | ***P*** |
| --- | --- | --- | --- | --- | --- | --- |
| **Categorical 25(OH)D** |  |  |  |  |  |  |
| Normal | ref |  | ref |  | ref |  |
| Low | 1.65 (1.17 to 2.36) | 0.005 | 1.26 (1.07 to 1.50) | 0.007 | 1.48 (1.26 to 1.74) | <0.001 |
| **Continuous 25(OH)D** |  |  |  |  |  |  |
| Per 10 nmol/L increment | 0.80 (0.73 to 0.88) | <0.001 | 0.94 (0.91 to 0.97) | <0.001 | 0.95 (0.93 to 0.97) | <0.001 |

**Abbreviations:** CLHLS, Chinese Longitudinal Healthy Longevity Survey; ELSA, English Longitudinal Study of Ageing; HRS, Health and Retirement Study; 25(OH)D, 25-hydroxyvitamin D; OR, odds ratio; CI, confidence interval.

**Note:** The outcome was frailty at baseline, defined as a frailty index ≥0.25. For categorical 25(OH)D, normal vitamin D status was used as the reference group. For continuous 25(OH)D, ORs represent the odds of frailty associated with each 10 nmol/L increment in serum 25(OH)D. Models were adjusted for age, sex, education, marital status, income, body mass index, smoking, drinking, diabetes, hypertension, and physical activity. The CLHLS and ELSA models were additionally adjusted for season of blood collection.

**Table S5 Cross-sectional associations of baseline serum 25(OH)D concentrations with frailty index across the CLHLS, ELSA, and HRS among participants with complete covariate data**

| **Serum 25(OH)D** | **CLHLS (N = 1,741) β (95% CI)** | ***P*** | **ELSA (N = 4,984) β (95% CI)** | ***P*** | **HRS (N = 4,983) β (95% CI)** | ***P*** |
| --- | --- | --- | --- | --- | --- | --- |
| **Categorical analysis** |  |  |  |  |  |  |
| Normal | Ref |  | Ref |  | Ref |  |
| Low | 0.013 (0.005 to 0.022) | 0.002 | 0.011 (0.005 to 0.017) | <0.001 | 0.021 (0.012 to 0.030) | <0.001 |
| **Continuous analysis** |  |  |  |  |  |  |
| Per 10 nmol/L increment | −0.006 (−0.008 to −0.004) | <0.001 | −0.003 (−0.004 to −0.002) | <0.001 | −0.002 (−0.003 to −0.001) | <0.001 |

**Note:** Analyses were restricted to participants with complete covariate data. Normal 25(OH)D concentrations were defined as >50 nmol/L and low concentrations as ≤50 nmol/L. Models were adjusted for age, sex, education, marital status, income, body mass index, smoking, drinking, diabetes, hypertension, and physical activity; the CLHLS and ELSA were additionally adjusted for season of blood collection. CLHLS, Chinese Longitudinal Healthy Longevity Survey; ELSA, English Longitudinal Study of Ageing; HRS, Health and Retirement Study; 25(OH)D, 25-hydroxyvitamin D; CI, confidence interval.

**Figure S5** Restricted cubic spline analyses of the associations of 25(OH)D concentrations with FI and incident frailty (excluding participants with missing covariate data). (A) Association between 25(OH)D concentrations and FI in CLHLS. (B) Association between 25(OH)D concentrations and FI in ELSA. (C) Association between 25(OH)D concentrations and FI in HRS. (D) Association between 25(OH)D concentrations and incident frailty in CLHLS. (E) Association between 25(OH)D concentrations and incident frailty in ELSA. (F) Association between 25(OH)D concentrations and incident frailty in HRS. Adjusted for age, sex, education, marital status, income, body mass index, smoking, drinking, diabetes, hypertension and physical activity, the CLHLS and ELSA were additionally adjusted for season. Abbreviation: CLHLS: Chinese Longitudinal Healthy Longevity Survey; ELSA: English Longitudinal Study of Ageing; HRS: Health and Retirement Study; FI: frailty index.

**Figure S6** Sensitivity analyses of the associations of 25(OH)D concentrations (Normal/lower) with incident frailty in CLHLS, ELSA and HRS. (A) Overview of the sensitivity analysis framework. (B) Sensitivity analysis results for CLHLS. (C) Sensitivity analysis results for ELSA. (D) Sensitivity analysis results for HRS. Sensitivity analyses included analyses restricted to participants with complete covariate data, analyses restricted to those with at least two follow-up assessments, a 2-year lag analysis, competing-risk models, discrete-time models, and analyses using an alternative FI cut-off to define frailty. Adjusted for age, sex, education, marital status, income, body mass index, smoking, drinking, diabetes, hypertension and physical activity, the CLHLS and ELSA were additionally adjusted for season. Abbreviation: CLHLS: Chinese Longitudinal Healthy Longevity Survey; ELSA: English Longitudinal Study of Ageing; HRS: Health and Retirement Study; FI: frailty index; CI: confidence intervals; HR: hazard ratios.

**Table S6. Comparison of the components and operational definitions used to construct the frailty index across the CLHLS, ELSA, and HRS**

| **Domain** | **Deficit** | **CLHLS** | **ELSA** | **HRS** |
| --- | --- | --- | --- | --- |
| **General health** | Self-rated health | Very good 0; good .25; okay .5; bad .75; very bad 1 | Poor self-rated general health | Poor self-rated general health |
|  | Interviewer-rated health | Healthy 0; moderately ill .5; very ill 1 | — | — |
| **Psychological** | Fear/anxiety | Frequency of feeling fearful/anxious, 0–1 | — | — |
|  | Feeling useless with age | Frequency, 0–1 | — | — |
|  | Positive outlook | “Look on the bright side”; frequency, 0–1 | — | — |
|  | Emotional/psychiatric problems | — | Emotional, nervous, or psychiatric problem | Emotional, nervous, or psychiatric problem |
|  | Depressive symptoms | — | CESD-8 ≥4 | CESD-8 ≥4 |
| **Cognitive/neurological** | Dementia/memory disorder | Dementia | Alzheimer’s disease, dementia, senility, or serious memory impairment | Alzheimer’s disease, dementia, senility, or serious memory impairment |
|  | Cognition | — | Memory + orientation deficit score, 0–1 | Memory + orientation deficit score, 0–1 |
|  | Parkinson’s disease | Parkinson’s disease | — | — |
| **Sensory** | Vision | Visual performance: normal 0; impaired .5–1 | Fair/poor self-rated eyesight | Fair/poor self-rated eyesight |
|  | Hearing | Normal 0; partial/hearing aid .5; unable 1 | Fair/poor self-rated hearing | Fair/poor self-rated hearing |
|  | Cataract | Cataract | — | — |
|  | Glaucoma | Glaucoma | — | — |
| **ADL** | Bathing | Independence/assistance required, 0–1 | Difficulty bathing/showering | Difficulty bathing/showering |
|  | Dressing | Independence/assistance required, 0–1 | Difficulty dressing | Difficulty dressing |
|  | Toileting | Independence/assistance required, 0–1 | Difficulty using toilet | Difficulty using toilet |
|  | Transfer/bed mobility | Independence/assistance required, 0–1 | Difficulty getting in/out of bed | Difficulty getting in/out of bed |
|  | Eating | Independence/assistance required, 0–1 | Difficulty eating | Difficulty eating |
|  | Continence | Independence/assistance required, 0–1 | — | — |
| **IADL** | Managing money | — | Difficulty managing money | Difficulty managing money |
|  | Taking medication | — | Difficulty taking medication | Difficulty taking medication |
|  | Shopping | — | Difficulty shopping for groceries | Difficulty shopping for groceries |
|  | Preparing meals | — | Difficulty preparing meals | Difficulty preparing meals |
|  | Housework | Frequency of housework, 0–1 | — | — |
|  | Making decisions | Frequency of making own decisions, 0–1 | — | — |
|  | Keeping belongings tidy | Frequency, 0–1 | — | — |
| **Mobility/physical function** | Walking | Ability to walk 1 km, 0–1 | Difficulty walking 100 yards | Difficulty walking one block |
|  | Rising from chair | Without hands 0; with hands .5; unable 1 | Difficulty rising after prolonged sitting | Difficulty rising after prolonged sitting |
|  | Stair climbing | — | Difficulty climbing several flights without resting | Difficulty climbing several flights without resting |
|  | Lifting/carrying | Ability to carry 5 kg, 0–1 | Difficulty lifting/carrying >10 lb | Difficulty lifting/carrying >10 lb |
|  | Stooping/kneeling/crouching | — | Difficulty stooping/kneeling/crouching | Difficulty stooping/kneeling/crouching |
|  | Reaching overhead | — | Difficulty reaching above shoulder | Difficulty reaching above shoulder |
|  | Picking up object | Pick up book from floor: standing 0; sitting .5; unable 1 | Difficulty picking up 5-pence coin | Difficulty picking up a dime |
|  | Upper-body flexibility | Hands behind neck, 0–1 | — | — |
|  | Upper-body flexibility | Hands behind lower back, 0–1 | — | — |
|  | Turning/balance | ≥6 steps for 360° turn = 1 | — | — |
|  | Chopstick use | Unable to use chopsticks = 1 | — | — |
| **Cardiovascular/metabolic** | Hypertension | Hypertension | Hypertension | Hypertension |
|  | Diabetes | Diabetes | Diabetes | Diabetes/high blood sugar |
|  | Heart disease | Heart disease | — | — |
|  | Stroke/CVD | Stroke or cerebrovascular disease | — | — |
|  | Heart rate | ≥80 beats/min = 1 | — | — |
| **Other chronic diseases** | Cancer | Cancer | Cancer/malignant tumour | Cancer/malignant tumour |
|  | Arthritis | — | Arthritis | Arthritis/rheumatism |
|  | Chronic respiratory disease | Bronchitis, emphysema, pneumonia, or asthma | Chronic lung disease | Chronic lung disease |
|  | Tuberculosis | Tuberculosis | — | — |
|  | Gastric/duodenal ulcer | Gastric or duodenal ulcer | — | — |
|  | Bedsore | Bedsore | — | — |
|  | Prostate tumour | Prostate tumour | — | — |
| **Recent morbidity** | Serious illness | Serious illness during previous 2 years | — | — |

**Abbreviations:** FI, frailty index; CLHLS, Chinese Longitudinal Healthy Longevity Survey; ELSA, English Longitudinal Study of Ageing; HRS, Health and Retirement Study; ADL, activities of daily living; IADL, instrumental activities of daily living; CESD-8, eight-item Center for Epidemiologic Studies Depression Scale; CVD, cerebrovascular disease.

**Table S7. Distribution of blood collection season across the CLHLS and ELSA cohorts**

| **Season** | **CLHLS, n (%)** | **ELSA, n (%)** |
| --- | --- | --- |
| Spring | 524 (25.7%) | 462 (7.6%) |
| Summer | 1,392 (68.3%) | 1,424 (23.5%) |
| Autumn | 122 (6.0%) | 2,566 (42.3%) |
| Winter | — | 1,609 (26.5%) |

**Abbreviations:** CLHLS, Chinese Longitudinal Healthy Longevity Survey; ELSA, English Longitudinal Study of Ageing;

**Table S8. Cross-sectional associations of baseline serum 25(OH)D concentrations with frailty index among adults aged ≥65 years in ELSA and HRS**

| **Serum 25(OH)D** | **ELSA (n = 3332),** **β (95% CI)** | ***P*** | **HRS (n = 5031), β (95% CI)** | ***P*** |
| --- | --- | --- | --- | --- |
| **Categorical 25(OH)D** |  |  |  |  |
| Normal | ref |  | ref |  |
| Low | 0.013 (0.005 to 0.021) | 0.002 | 0.022 (0.013 to 0.031) | <0.001 |
| **Continuous 25(OH)D** |  |  |  |  |
| Per 10 nmol/L increment | -0.004 (-0.006 to -0.002) | <0.001 | -0.003 (-0.004 to -0.002) | <0.001 |

**Abbreviations:** ELSA, English Longitudinal Study of Ageing; HRS, Health and Retirement Study; 25(OH)D, 25-hydroxyvitamin D; OR, odds ratio; CI, confidence interval.

**Note:** The outcome was frailty at baseline, defined as a frailty index ≥0.25. For categorical 25(OH)D, normal vitamin D status was used as the reference group. For continuous 25(OH)D, ORs represent the odds of frailty associated with each 10 nmol/L increment in serum 25(OH)D. Models were adjusted for age, sex, education, marital status, income, body mass index, smoking, drinking, diabetes, hypertension, and physical activity. The ELSA models were additionally adjusted for season.

**Table S9. Cross-sectional associations of serum 25(OH)D with prevalent frailty at baseline among adults aged ≥65 years in ELSA and HRS**

| **Serum 25(OH)D** | **ELSA (n = 3332), OR (95% CI)** | ***P*** | **HRS (n = 5031), OR (95% CI)** | ***P*** |
| --- | --- | --- | --- | --- |
| **Categorical 25(OH)D** |  |  |  |  |
| Normal | ref |  | ref |  |
| Low | 1.27 (1.04 to 1.56) | 0.020 | 1.48 (1.26 to 1.74) | <0.001 |
| **Continuous 25(OH)D** |  |  |  |  |
| Per 10 nmol/L increment | 0.96 (0.92 to 0.99) | 0.032 | 0.95 (0.93 to 0.97) | <0.001 |

**Abbreviations:** ELSA, English Longitudinal Study of Ageing; HRS, Health and Retirement Study; 25(OH)D, 25-hydroxyvitamin D; OR, odds ratio; CI, confidence interval.

**Note:** The outcome was frailty at baseline, defined as a frailty index ≥0.25. For categorical 25(OH)D, normal vitamin D status was used as the reference group. For continuous 25(OH)D, ORs represent the odds of frailty associated with each 10 nmol/L increment in serum 25(OH)D. Models were adjusted for age, sex, education, marital status, income, body mass index, smoking, drinking, diabetes, hypertension, and physical activity. The ELSA models were additionally adjusted for season.

**Table S10. Longitudinal associations of baseline serum 25(OH)D concentrations with incident frailty among adults aged ≥65 years in ELSA and HRS**

|  | **ELSA** |  | **HRS** |  |
| --- | --- | --- | --- | --- |
| **Categorical 25(OH)D** | **HR (95%CI)** | ***P*** | **HR (95%CI)** | ***P*** |
| Normal | ref |  | ref |  |
| Low | 1.29 (1.06 to 1.56) | 0.010 | 1.04 (0.85 to 1.27) | 0.695 |
| **Continuous 25(OH)D** | **-** | **-** | **-** | **-** |
| Per 10 nmol/L | 0.95 (0.91 to 1.00) | 0.031 | 0.98 (0.95 to 1.00) | 0.037 |

**Abbreviations:** ELSA, English Longitudinal Study of Ageing; HRS, Health and Retirement Study; 25(OH)D, 25-hydroxyvitamin D; HR, hazard ratio; CI, confidence interval.

**Note:** Longitudinal analyses were restricted to participants who were non-frail at baseline. Normal 25(OH)D concentrations were defined as >50 nmol/L and low concentrations as ≤50 nmol/L. Continuous associations were estimated per 10 nmol/L increment in baseline serum 25(OH)D. Models were adjusted for age, sex, education, marital status, income, body mass index, smoking, drinking, diabetes, hypertension, and physical activity; the ELSA were additionally adjusted for season.
